# Liquid Phase TEM of Diffusing Emulsion Droplets

**DOI:** 10.1002/smll.202512006

**Published:** 2026-01-30

**Authors:** Maria A. Vratsanos, Evangelos Bakalis, Chiwoo Park, Francesco Zerbetto, Nathan C. Gianneschi

**Affiliations:** ^1^ Department of Materials Science & Engineering Northwestern University Evanston Illinois USA; ^2^ Department of Chemistry “G. Ciamician” Universita di Bologna Bologna Italy; ^3^ Department of Industrial and Manufacturing Engineering Florida State University Tallahassee Florida USA; ^4^ International Institute for Nanotechnology, Chemistry of Life Processes Institute Northwestern University Evanston Illinois USA; ^5^ Department of Chemistry, Department of Biomedical Engineering, Department of Pharmacology Northwestern University Evanston Illinois USA

**Keywords:** dynamics, emulsions, in situ transmission electron microscopy, random walks on fractals

## Abstract

The origin of the viscoelastic behavior that many nanoparticles display during diffusive motion is unknown. Such dynamics are difficult to record without sophisticated methods that combine a suitable observation window of motion in time with high image resolution. Herein, we study and describe the diffusion of two types of particles in the form of emulsion droplets in situ via liquid phase TEM. For both, the observed particle motion in solution is anomalous (non‐Brownian) and is either sub‐ or super‐diffusive. Fractional Brownian motion (fBm) and random walks on fractals (RWF) are the two potential mechanisms. It can be challenging to differentiate these since they may have the same position or velocity autocorrelation function, but they diverge in the average number of sites visited, which is connected to the fractal dimension of the walk. We conclude that droplet‐surface interactions and electron beam fluence create a fractal energy landscape yielding peculiar dynamics.

## Graphical abstract

Motion of emulsion droplets was observed via in situ liquid phase transmission electron microscopy. Analysis revealed that the motion is self‐affine and influenced by multiple stochastic processes, as well as a fractal landscape created by the electron beam. The largest droplets exhibited superdiffusional motion as a result of spatial constraints, while smaller droplets exhibited random walks on fractals.

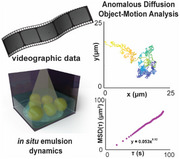

## 1 Introduction

Since the advent of electron microscopy, the observation of in situ materials and processes has been a fundamental goal. Liquid phase transmission electron microscopy (LPTEM) has come to the forefront over the past decade with recent developments in material fabrication techniques and technologies enabling researchers to seal liquid samples between electron transparent membranes, including silicon nitride (SiN_x_) and graphene, against the high vacuum environment of the microscope [1, 2, 3, 4]. Corresponding advances in detectors have enabled visualization and image acquisition for such systems with increasing spatiotemporal resolution [5, 6, 7, 8, 9, 10, 11]. While these innovations have enabled direct observation of solvated reactions [12, 13, 14], morphology [15, 16, 17, 18, 19], and assembly pathways [20, 21, 22, 23, 24, 25, 26, 27], fundamental questions regarding the nature of in situ dynamics remain. In principle, observing the motion of nanoparticles should be a straightforward experiment – simply load the liquid cell with nanoparticles, turn on the beam and record. However, a variety of factors result in anomalous observed motion. These include confinement effects, solvent ordering at the SiN_x_ interface, charging of the SiN_x_ membrane by the e‐beam, local chemopotential gradients resulting from solvent radiolysis and more [28, 29, 30, 31, 32, 33, 34, 35, 36, 37, 38, 39, 40, 41]. Many of these downstream radiolytic effects will also be exacerbated by the choice of sample; metallic nanoparticles will act as local dose enhancers, accelerating radiolysis in their vicinity, while radical scavengers and choice of liquid cell format can provide protection [42, 43, 44, 45]. These deleterious effects are balanced with the fact that high Z contrast inorganic nanoparticles are appealing for tracking and identification in the inherently low contrast confines of the liquid cell and as such they have been the focus of much of the study in this area [4, 28, 29, 30, 31, 33, 34, 35, 39, 40, 46, 47].

The studies presented herein are motivated by a desire to elucidate how in situ diffusivity is altered from the bulk and to define the type of motion observable and affected by confinement. Diffusion of molecular and ionic species is clearly occurring in LPTEM because diffusion‐limited nucleation and growth processes have been observed [14, 21, 22, 48, 49, 50, 51, 52]. However, if the motion of larger, nanoscale species is significantly hindered by spatial confinement and the effects highlighted above, these must be considered when employing in situ observations to make quantitative conclusions about the rate at which observed processes are occurring. Essentially, we need to identify and quantify the discrepancy from the bulk and apply such findings to the observed processes to reconcile them. If it is possible to routinely observe unhindered motion, then answering more fundamental questions about transport and growth kinetics becomes possible. If it is not, it becomes clear that experimental results from LPTEM need to be viewed through a more critical lens if nanoparticle diffusion is required or is the process under study [28]. In these cases, additional caveats must be included when extrapolating from the liquid cell to bulk solution. The results of this study provide a more complete understanding of how the environment of the liquid cell deviates from equilibrium without the confounding influence of metallic nanoparticles. This enhanced understanding will permit a more nuanced view of in situ dynamics by enabling the disentanglement of beam‐induced dynamics from the processes being studied.

We propose that to effectively probe diffusion in the liquid cell, it is necessary to consider the motion of low contrast, less e‐beam reactive materials that have minimal interaction with both the electron beam and the SiN_x_ interface. Systems of colloidal emulsion droplets seem an ideal candidate, as they have been shown to yield exceptional contrast via LPTEM, exhibit size control and have minimal interactions with the SiN_x_ membrane [19].

## 2 Results and Discussion

### 2.1 Experimental Details

The low contrast expected for emulsions necessitated advanced image processing techniques, as they are of insufficient contrast for typical segmentation algorithms. Image acquisition parameters were estimated a priori based upon estimated droplet size, liquid cell thickness and signal constraints (see Sections SI–SV for materials selection, sample preparation, liquid cell assembly, microscopy set up and estimation of required resolution). Further, we demonstrate in this analysis that the spatial constraints of the liquid cell perturb the system prior to the consideration of any e‐beam effects and thus can be considered far from equilibrium.

Perfluorohexane (PFH) was emulsified in water using Capstone FS‐30, a commercial fluorosurfactant. Given the excellent contrast of the PFH, droplets were clearly visible without additional processing; thus, videos were recorded using a screen capture software at lower spatial resolution (1k instead of 4k) to reduce the computational load of storing and processing these data. Once acquired, videos were of sufficient contrast to be segmented using the TrackMate plugin, which yields droplet location and size for each frame [53, 54].

**FIGURE 1:**
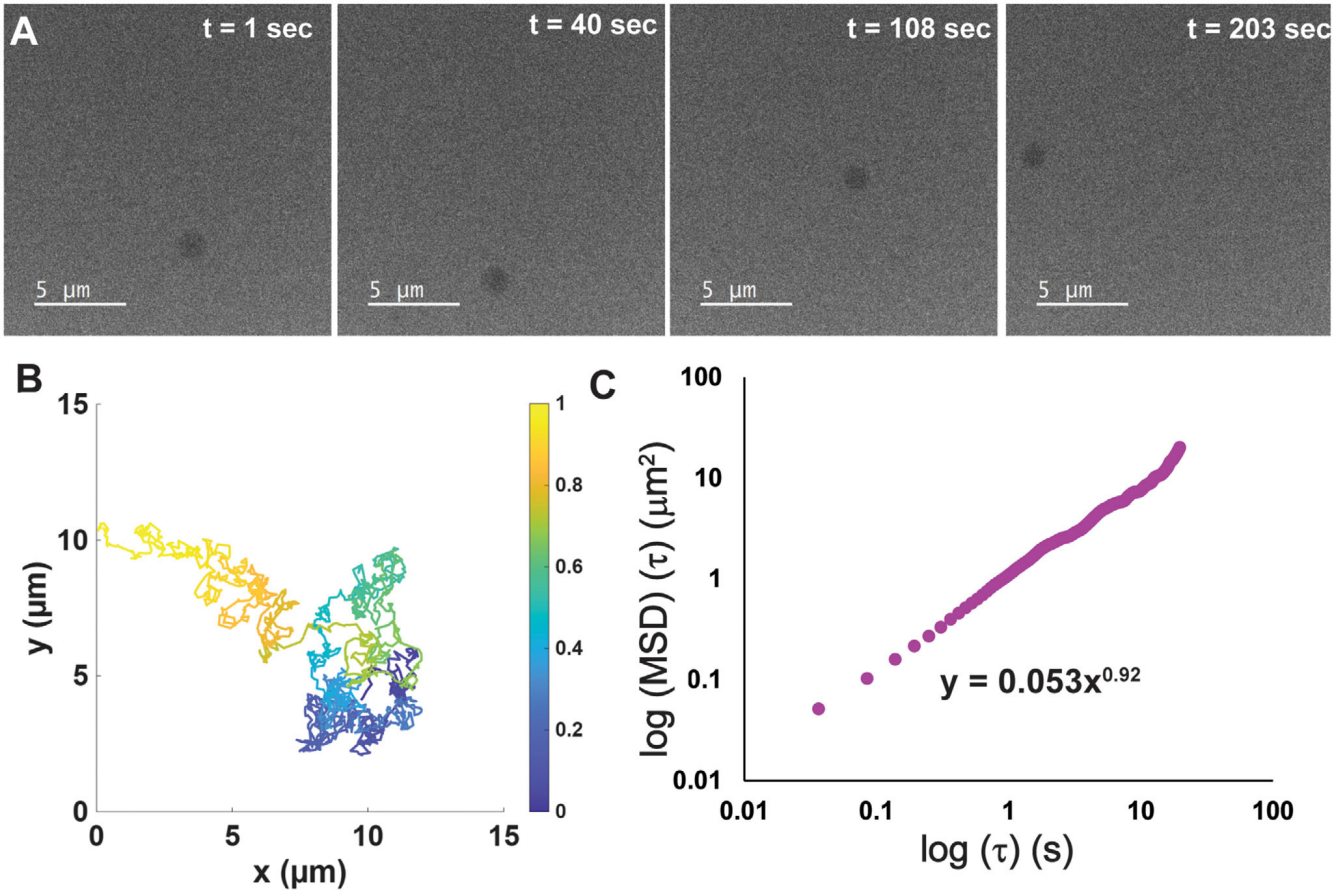
Motion overview and analysis for perfluorohexane droplet PFH_1. (A) Selected micrographs from videographic data at indicated time points showing displacement of the droplet in time. (B) Plot of overall droplet trajectory with coloration indicating time. Colors range from black to yellowas time progresses. (C) Graph of mean square displacement over time plotted logarithmically. Power law fit of data is displayed on plot.

After individual droplet trajectories were determined over the entire video, the mean square displacement was calculated and plotted with respect to τ to yield an equation of the form *MSD* = 4D*τ^*n*^, giving both the diffusivity constant (D) and the exponent (n). The exponent offers a preliminary categorization of the droplet's motion character (*n* = 1 for Brownian, and *n* < 1 or *n* > 1 for sub‐ and superdiffusion, respectively) [38, 55]. Given the ease with which PFH trajectories were extracted, and with their relative length, we chose to first analyze those datasets (Figure 1).

### 2.2 Statistical Analysis

The data has not undergone any preprocessing and all results are presented with the computed error. All data and sample sizes are presented in Table S2 of the Supporting Information material. For the fractal analysis, custom codes were used (see below).

Here, we observed dark droplets moving for several minutes at a time (Figure 1A, Video S1), yielding a robust trajectory for analysis (Figure 1B). Upon analysis of the MSD, the power law fit indicates motion of a quasi Brownian nature (exponent ≈ 1) (Figure 1C).[56, 57] Experimentally measured diffusivities for droplets of differing radii were inversely proportional to droplet size, as is consistent with theory.

### 2.3 Methodology of Motion Analysis

**FIGURE 2:**
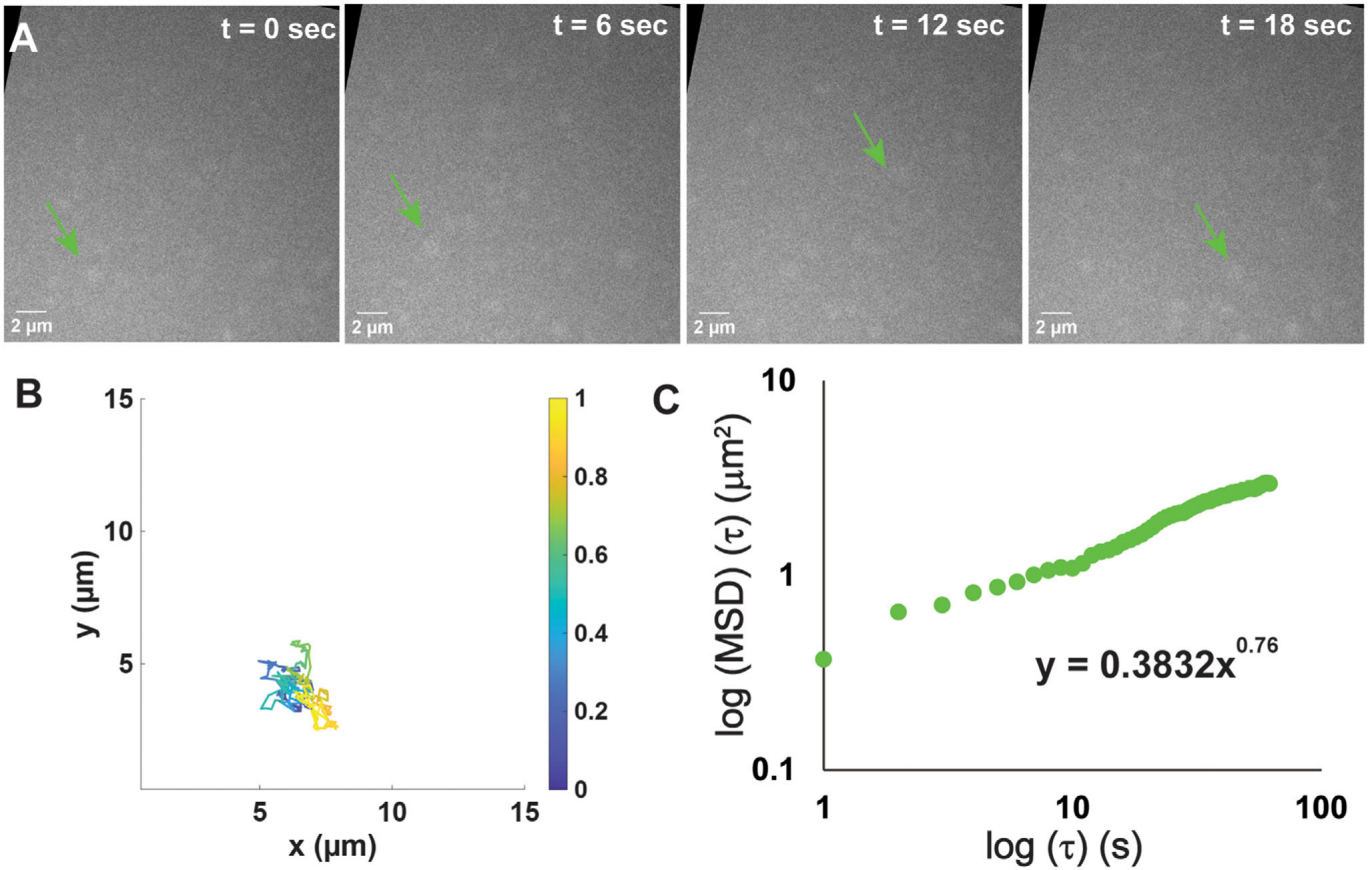
Motion overview and analysis for a representative isooctane droplet (Iso_1). (A) Selected micrographs from videographic data at indicated time points showing motion of droplet denoted by the green arrow over time. (B) Plot of overall droplet trajectory, with coloration indicating time. Colors range from black to yellow as time progresses. (C) Graph of mean square displacement over time, plotted logarithmically. Power law fit of data is displayed on plot.

While the PFH droplets have superior contrast, isooctane droplets could be produced and observed in greater populations as demonstrated in previous studies (Video S2) [19]. Here, the droplets were emulsified with Aerosol OT (AOT) as in previous work. However, the number of droplets, coupled with their low contrast, makes the extraction of trajectories more difficult. Therefore, we employed the multi‐object tracking algorithm [58, 59]. The algorithm is capable of handling tracking complexities due to image artifacts and temporary particle disappearances for some image frames to accurately identify the trajectories. The method adopts a robust statistics approach to identify and subtract the image background due to uneven illumination and background material, and each of the remaining foreground images are fitted by an ellipse model to identify elliptical boundaries of particles. Once extracted, we analyzed these trajectories (Figure 2). Sustained motion was again observed (Figure 2A, B). However, this motion was found to be significantly sub‐diffusive (Figure 2C, D, Tables S2–S4).

When we consider the trajectories of isooctane droplets with generic tracking tools such as TrackPy, experimental diffusivities are found to be comparable or greater than the prediction of Stokes‐Einstein, which is counter to expectation and to observations of fluorinated oils, and is an inherently non‐physical result. This discrepancy could be the result of e‐beam induced localized heating, which would artificially reduce solvent viscosity in the area of study, or could be the result of deviating from models used to predict diffusivity, which were based on rigid polystyrene spheres much smaller than their environment, and therefore may differ from this situation where the droplets are compressible and could be similar to if not larger than the liquid cell thickness [60]. It is likely that both sources contribute to this discrepancy. Thus, subsequent analysis relied on the method of anomalous diffusion object‐motion analysis (or ADOMA), which considers the contribution of additional moments, q ∈ (0, 4], including fractional values and constructing the structure function z(q); notice that for *q* = 2 the method provides the MSD. Details of the method have been given elsewhere [38]. (Sections SX–SXI). The shape of the structure function is a strong descriptor for the classification of random motion. A linear dependence of z(q) versus q underlines a quasi‐Gaussian behavior and potentially points to fractional Brownian motion (fBm) ‐ be it sub/super diffusive ‐ driven by fractional Gaussian noise (fGn). The slope of the line is half of the MSD exponent and is called the Hurst exponent; its value is 0.5 for Brownian motion. When the shape of the structure function departs from linearity, its description is split into a linear term or monofractal term and a multifractal correction [38].

The analyzed data sets have the ordered form {x_i_, y_i_}, where x,y refer to the x− and y− axes of the laboratory frame of reference or field of view (FoV). The index i provides the number of measurements and is connected to the elapsed time, t = i × τ. We also create the sequence $l_{i}=\sqrt{x_{i}^{2}+y_{i}^{2}}$, which stands for the lateral displacement of the probe particle on the FoV. All *x_i_*, *y_i_*, and *l_i_* have been analyzed for trajectories with at least 100 points (Table S1). A linear form of the structure function cannot by itself fully assign a random motion as fBm. It needs to be confirmed by the form of the velocity auto‐correlation function and the excess kurtosis, which for quasi‐Gaussian processes should tend to zero. Moreover, a linear dependence of z(q) for the lateral motion, when not supported by linear forms for the corresponding structure functions along x‐ and y‐axes, can reflect coupling of movements that are the result of environmental constraints. The presence of these constraints leads to sophisticated random walks, which retain their Gaussian nature; see the behavior of isooctane droplets.

The motion of PFH and isooctane droplets are significantly different from one another, likely because of the constraints imposed by the liquid cell. Indeed, the PFH droplets analyzed here have radii of 750 and 2000 nm, respectively, which is much larger than the thickness of the cell (500 nm) and are therefore squeezed between the membranes of the cell. On the other hand, the radii of isooctane droplets are in the range 150–500 nm, comparable or smaller than the thickness of the cell.

### 2.4 Analysis of Perfluorohexane Droplets

**FIGURE 3:**
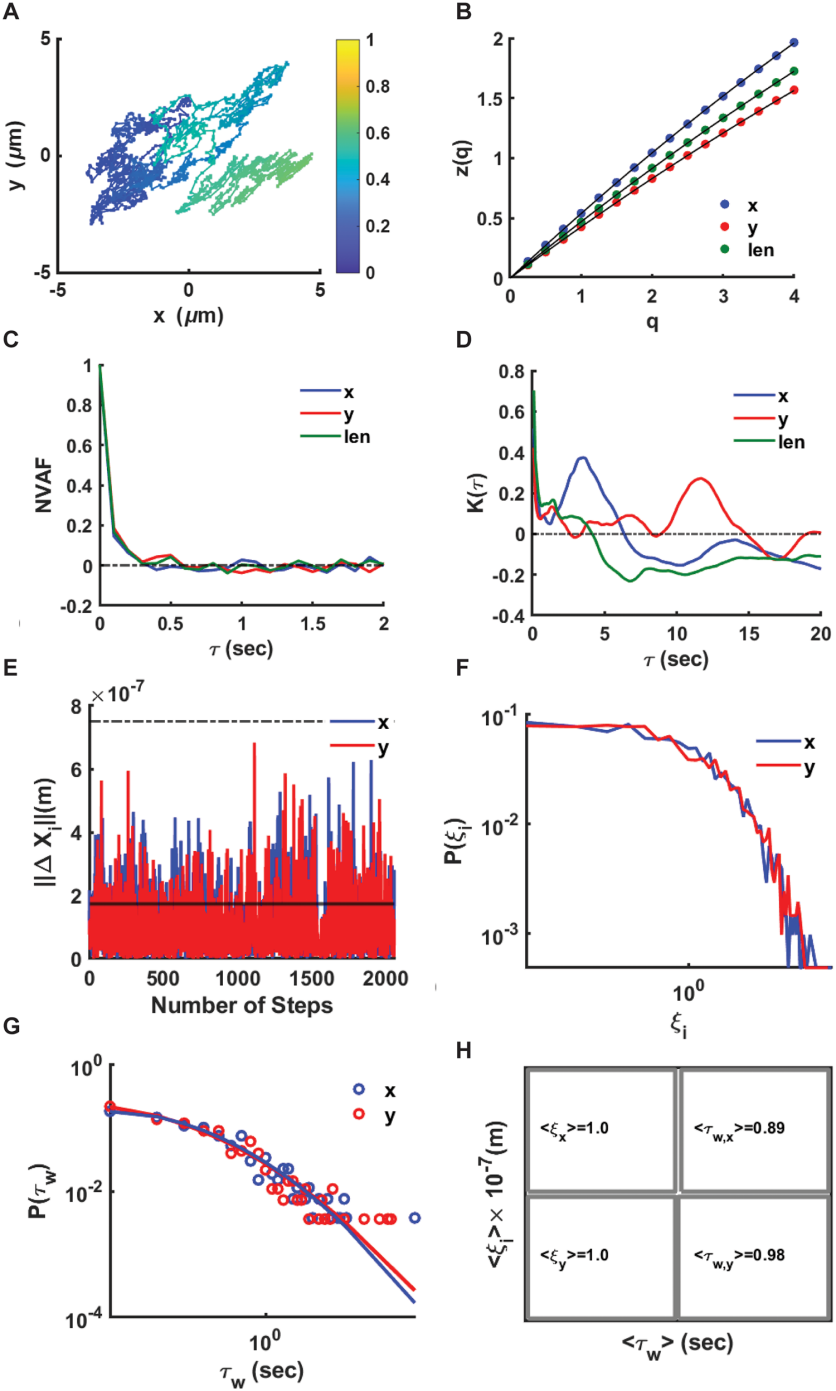
ADOMA results for PFH_1. (A) Motion trajectory of droplet PFH_1 (*r* = 750 nm). Colors range from blue to yellow as time progresses. (B) Structure functions. (C) Normalized velocity autocorrelation functions. (D) Excess kurtosis. (E) Increments along x‐ and y‐axes. (F) Probability distribution of ξ_i_=||ΔX_i_||/<||ΔX_i_||>. (G) Probability distribution of waiting times, whereas the value ξ_i_=2s (s is the standard deviation) has been set as cut‐off distance. (H) Mean of the length taken along the x‐ and y‐ axes and the mean of the waiting time.

Figure 3 shows the analysis for PFH droplet PFH_1 (Video S2). The recording time is approximately 3.42 min (minimum time lag of 0.1 sec) and the center of mass of the droplet explores a maximum surface area of about ≈60 µm^2^ (Figure 3A). Based only on the exponent of the second moment, one classifies the motion as Brownian along the x‐axis (n_x_ ≈1.04), sub‐diffusive along the y‐axis (n_y_ ≈0.83) and slightly sub‐Brownian for lateral motion (*n* = 0.92). Such a classification is in line with the values of z(*q* = 1) and z(*q* = 2) along x‐ and y‐axes as well for lateral motion because of the relation z(*q* = 2) = 2z(*q* = 1) (Figure 3B, Table S2). However, this characterization is challenged twice; by the presence of a convex shape of structure functions and by the form of the normalized velocity autocorrelation function (NVAF) (Figure 3C). If motion were Brownian, the NVAF (blue line) would be delta correlated, while if motion were sub‐diffusive fBm (red and green lines), then a distinguishable minimum at negative values would exist [61]. By contrast, NVAF retains a memory of about three to four steps (0.3 to 0.4 sec), then starts to fluctuate around zero. The excess kurtosis deviates from zero (Figure 3D). For short time series, quasi‐Gaussian processes can have excess kurtosis that fluctuate in the range [−1,1] [62]. Overall, two out of three of the descriptors rule out fractional Brownian motion and Brownian motion as motion types.

At the time scale of the observation, the departure from Gaussian behavior signals the existence of droplet‐membrane and droplet‐fluid interactions. The lengths of steps ||ΔX_i_|| along x‐ and y‐axes are consistently much smaller than the droplet radius of (≈7.5 × 10^−7^ m) (Figure 3E) and their mean values are 0.996 × 10^−7^ /1.031 × 10^−7^ m in x/y‐axes respectively, with standard deviation of 0.880 × 10^−7^ m for both axes. Note that a handful of events exists where step‐length is about the half of the radius.

The probability distribution of the dimensionless variable ξ_i_ = ||ΔX_i_||/<||ΔX_i_||> (Figure 3F) confirms the existence of tails, making clear that the droplet undertakes very small steps or slips and that to take a large step or move a certain distance, a barrier must be overcome. If a cut‐off distance of twice the standard deviation is considered, we obtain the probability distribution of waiting times, or how frequently the droplet undertakes steps either in x‐ or in y‐axes whose length is larger than the cut‐off value. The distributions as well the best fits are displayed in Figure 3G. The log normal distribution works well for both axes, the parameters of the fit are listed in Table S5 and return mean waiting times <τ_w_> = 0.89 sec and 0.98 sec for x‐ and y‐axes, respectively. Assuming that the simplest form of transition state theory (TST) applies, and the mean waiting time is the reciprocal of the rate constant, k, then the energy barrier is provided by the formula $\Delta E=-k_{B}\mathrm{Tlog}\left(\frac{h}{k_{B}T\left\langle \tau_{w}\right\rangle }\right)$, where k_B_T is the Boltzmann's constant times the temperature of the sample and h is the Planck's constant. Considering T = 293.25 K and <τ_w_> = 0.89/0.98 returns a barrier of 17.1 kcal/mol in x‐axis and 17.15 kcal/mol in y‐axis.

The second PFH droplet, PFH_2 (Video S3) presents features of directional motion, which lead to super‐diffusion despite very small increments with respect to its radius. After reducing the cut‐off distance to one standard deviation, we found an equivalent barrier of about 16.5 kcal/mol, which is comparable to the findings for droplet PFH_1. The latter likely indicates the time needed for the re‐organization of droplet surfactants. The barrier either relates to binding interactions between the emulsion particles and the membrane of the liquid cell or could be due to a re‐organization from the current state of the droplet to an energy minimum (note that emulsions are metastable materials), or a combination of both. In either case, it is a chemical signature of the droplet motion associated with it being an emulsion droplet.

### 2.5 Analysis of Isooctane Droplets

**FIGURE 4:**
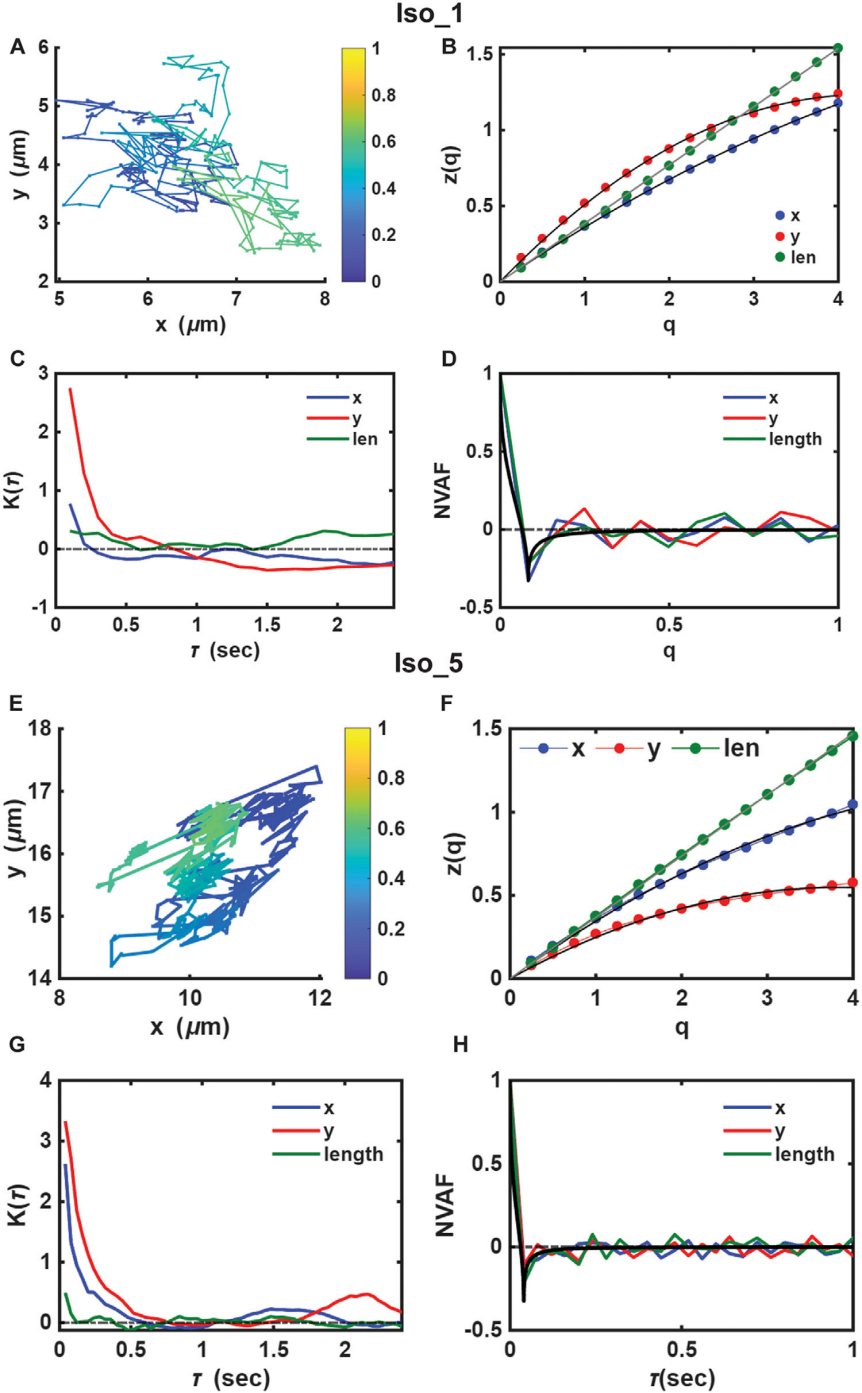
ADOMA analysis for isooctane droplets Iso_1 and Iso_5, the two longest recorded trajectories. (A) Iso_1 motion trajectory (colors range from blue to yellow as time progresses), (B) Iso_1 structure function z(q). (C) Iso_1 excess kurtosis K(τ). (D) Iso_1 normalized velocity autocorrelation function (NVAF) increments along x‐ and y‐axes. (E) Iso_5 motion trajectory (colors range from blue to yellow as time progresses). (F) Iso_5 structure function z(q). (G) Iso_5 excess kurtosis K(τ). (H) Iso_5 NVAF increments along x‐ and y‐axes. Black lines in Figure 4D and H stand for the theoretical prediction of NVAF if motion were fBm [61].

We can perform an analogous analysis of the isooctane droplet trajectories. Here, the results illustrate various properties of the two longest isooctane trajectories, Iso_1 and Iso_5 (Figure 4). The droplets have radii of 150 and 200 nm, respectively, and their motion has been recorded for greater than 20 seconds with minimum lags 0.083/0.04 sec, respectively. Both droplets exhibit very similar characteristics in their motion.

**FIGURE 5:**
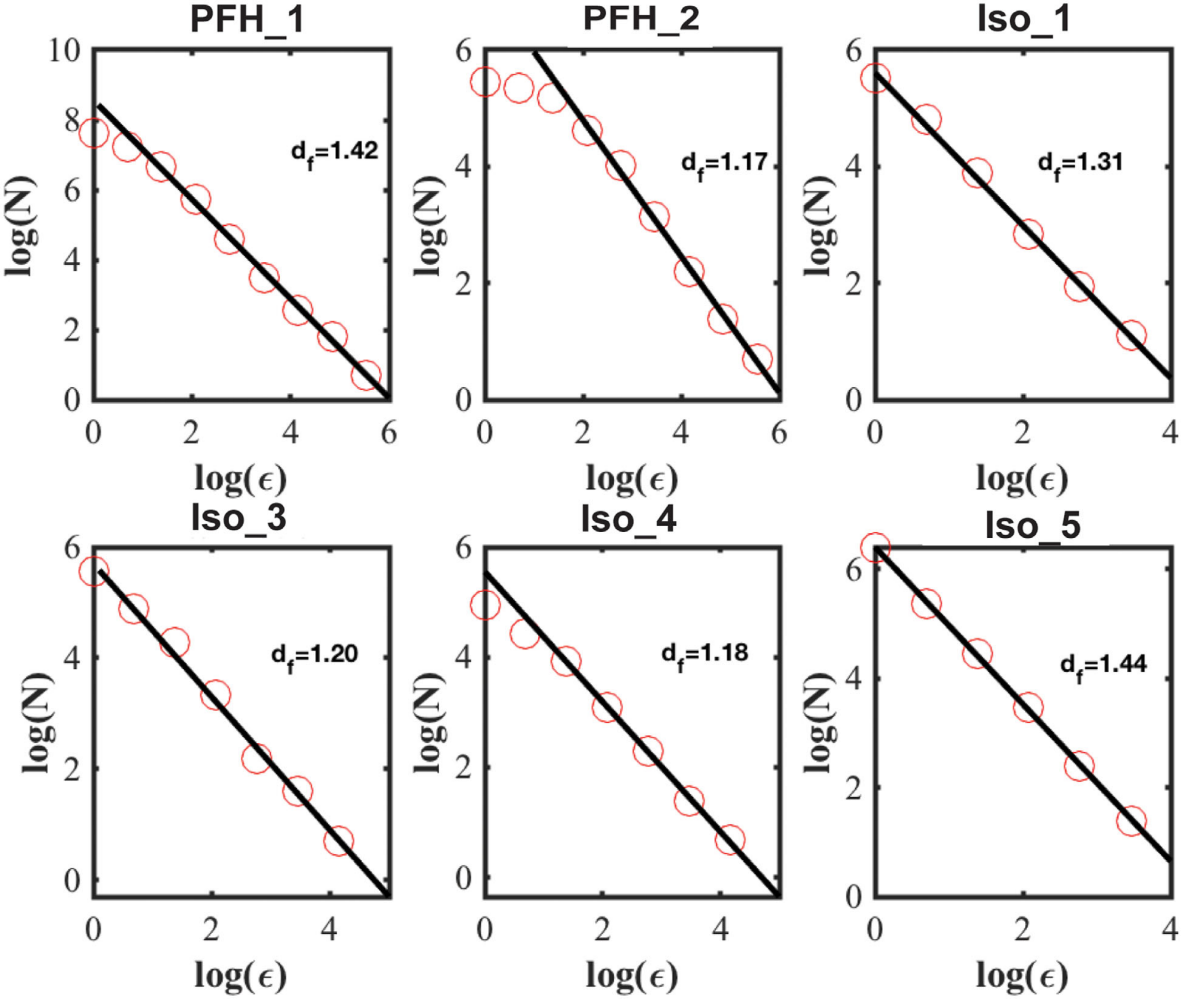
The slope of the number of squares (N) versus the square's length of the side, ϵ, in the log‐log plot returns the fractal dimension, d_f_ of the droplet's motion on the FoV. The values of d)_f_ with the corresponding error are: (1.42 ± 0.04), (1.17 ± 0.03), (1.31 ± 0.03), (1.20 ± 0.04), (1.18 ± 0.07), (1.44 ± 0.01). The fractal dimension of the walk of Iso_2, which is not depicted in this figure, is (1.32 ± 0.06).

Each of the droplets explores a small portion of the available surface and tends to scan continuously across a small subspace before moving with a somewhat longer step to an adjacent subspace (see for Figure 5 for the motion trajectories). The structure functions have convex shapes for motion in the y‐axis, it has linear or convex shape for motion in x‐axis and it is linear for lateral motion for 4 out of 5 isooctane droplets analyzed (Table S4). Structure functions suggest an intrinsic coupling of x‐ and y‐axes, which returns a linear structure function for lateral motion and thus the existence of a unique scaling at all time scales. Bearing in mind the values of the scaling exponents for the first and second moments as well as for the variance (Table S2), the process shows a sub‐diffusive behavior for most of them, which points to anti‐persistent random walks that under certain conditions can be classified as sub‐diffusive fBm driven by fGN. The form of the NVAF supports this type of motion (Figure 4D,H), where the experimental curve is quite close to the theoretical prediction, the solid black line.[61] If fGn were the type of noise, and given that fBm is of Gaussian type, the excess kurtosis (K(τ)) would be zero. Figure 4C and G show that for lateral motion, for both droplets, excess kurtosis can be considered zero, thereby confirming fGn as the driving mechanism of the droplet. The exception to the rule is droplet Iso_3, whose motion is slightly subdiffusive and close to normal.

### 2.6 Classifying the Motion

The different structure functions along x‐ and y‐axes demand closer scrutiny since this can be due to a random walk on a fractal (RWF). fBm and RWF might describe anomalous motion, either caused by a viscoelastic environment (fBm) or by geometrical constraints (RWF). Their differences are detected either by the probability density function (PDF) of the increments or by the mean number of visited sites up to time, t [60, 62]. On the other hand, the mean number of visited sites up to a certain time is related to the fractal dimension, *d_f_*, which is obviously smaller for RWF, given that two points on a surface are connected via limited routes in comparison to fBm, where all routes are available.

The fractal dimension quantifies the “irregularities” of random motion. The kind of data that is available determines how the fractal dimension is calculated. For instance, if we have a spatial coordinate in time, which is a 2D sequence, then the fractal dimension is given by *d_f_* = 2−*H*. In this result, we arrive either by the generalized moments method (4−2*d_f_* = 2*H*) or by the spectral dimension (5−2*d_f_* = 2*H* +1), where H is the Hurst exponent. If the data only contain spatial coordinates, then the fractal dimension, or Hausdorff‐Besicovitch dimension, is approximately obtained by applying the box‐counting method [63]. This metric is a geometrical property of the path within the surface where the motion evolves and it is not to be confused with dynamical properties of the motion like scaling exponents. For fBm and *H* < 0.5, or, in other words, for subdiffusive fBm, the fractal dimension is always 2 [64], which means that every path on a surface is accessible for motion. Random walks on fractals (RWF) with finite moments and distribution lengths typically follow subdiffusion since particle motion is slowed down by disorders that shrink the available paths on a surface. In fact, this is the case for most of the droplets analyzed in this work (see Tables S1, S2, and S5). Super diffusion on fractal paths is also possible [65]. Calculated fractal dimension *d_f_*, for all the droplets are given in Figure 5 (see also Section SXI).

Here, superdiffusion and RWF arise in experiments where the fundamental difference is the size of the droplet present in the aqueous medium on the SiN_x_ membrane. The system is strongly off‐equilibrium. However, the conditions are stationary since the e‐beam is constant in time. Fractal analysis shows that motion, for most of the examined cases in Table S5, along each one of the axes is multifractal and can even be ascribed to a Levy walk, as the distributions of the increments (Figure 3E; Figure S3E for PFH) suggest. These increments are not taken simultaneously and indicate that one of the directions is used as a pivot point for changing path. These displacements reveal the fractal character of the trajectories.

The perfluorohexane droplets have the largest diameters of all droplets used and in full formation are from three to eight times the size of the cell. The smaller of the two undergoes a quasi‐Brownian motion (exponent 0.92), while the other one undergoes superdiffusion (exponent 1.48) and both of them are random walks on fractals (see Table S5). Their ability to move under these conditions is likely due to their strongly hydrophobic nature, which exploits the energy provided by the experimental conditions to minimize the interaction with water. The droplet flees in search of hydrophobic regions. The bulging of the membrane, especially for the larger droplet, provides favored directions for diffusion.

The isooctane droplets present RWF. Their nature is still hydrophobic, although less markedly than PFH. The droplet is able to explore the surrounding environment, which is patterned by the beam (see above), more thoroughly.

The unifying picture that emerges is that the environmental conditions, that provide a stationary input of energy, are exploited differently depending on the hydrophobic character of the droplet.

The barriers generate channels in which the diffusing particle travels. Super diffusion has been reported in a two‐dimensional complex plasma [66] and in 2D Yukawa liquids [67]. In crowded environments, with a large concentration of barriers, the motion can be super diffusive for intermediate durations [68].

Analysis shows that the motion of the droplets is self‐affine in the time scale of the measurement, since the motion along the x‐ and y‐axes of the FoV scale differently, (Tables S1 and S2). Given that movements along x‐ and y‐axes are multifractals, there exist various mechanisms acting multiplicatively and shaping the overall motion. Such mechanisms are the bulging of the liquid cell at the center of the viewing window, the energetic landscape created by the e‐beam on the membrane, droplet cells interactions to name a few. In addition, the droplets are intrinsically metastable so that during their motion they can and do re‐organize. It is also possible that the e‐beam rearranges the energetic landscape.

Under this scenario, the role of the beam is essential since it can impose on‐the‐fly patterning of the surface charge and/or chemistry along on which the motion of the droplet takes place, favoring certain paths and prohibiting others. This notion is further supported by the range of dimensions that goes from 1.17 to 1.44, which include the following 2D fractals: Triflake (1.2619), 2D Cantor dust (1.2619), Apollonian gasket (1.3057), 5 circles inversion fractal (1.328), Minkowski Sausage (1.36521), and quadratic von Koch curve (1.4649). These structures share the presence of large empty spaces where a particle walking on them could not reconnoitre [63, 64]. The 2D fractals can arise through a relatively simple mechanism. Specifcially, the electron beam interacts with the aqueous medium and the SiN_x_ membrane and the flow of electrons creates charged/polarized sites. Their location depends on the chemistry of the sample and the pattern of electrons from the beams. As such, they can hardly form a regular pattern. The charged/polarized sites must then be surrounded by other sites that minimize the electrostatic energy. The position of the initial sites will be random. The randomness of the initially charged sites generates a terrain that is fractal. On the fractal pattern of charges there are regions that are allowed or forbidden to tread. Systematically varying the electron beam dose rate and the structure of the sample will provide quantitative insight into the process and it's general applicability, in the future. In another context, soap foams, with gradually changing bubble sizes, display rheology dynamics emergent from fractal geometry in the energy landscapes of many complex fluids [64].

## 3 Conclusions

Here, we have presented results for the motion of emulsion droplets in situ via liquid phase TEM. Fractal analysis shows that droplet dynamics are self‐affine and, on the timescale of the measurement, scale differently along the x‐ and y‐axes, which is attributed among other factors to the bulging of the SiN_x_ membranes of the top and bottom of the liquid cell. In general, the multifractality of the motion indicates the presence of at least two different stochastic mechanisms that act multiplicatively on the motion. Such mechanisms, in addition to bulging, are connected either to the metastability of the droplets or to the barriers resulting from the reorganization of positive and negative charges because of the e‐beam, or lastly to the interaction of the droplets with the membrane. Further, we note, that the dimension of the fractals approach those reported in entirely different contexts. That is, for materials with behaviors dependent on their rheological properties, connected to structural disorder and metastability [49]. The motion of the perfluorohexane droplets is RWF, while the superdiffusive character of the larger one reflects the influence of the highly spatial constraint of the in situ environment. In addition the motion of the smaller isooctane droplets is also consistent with a random walk on a fractal surface. We posit that the interfacial surfactants present in these systems permit this motion to occur in a less hindered fashion by creating a surface which is able to slip and slide across the SiN_x_ membrane with minimal pinning events. Such motion is modular based on droplet size. Further, we posit that the use of soft materials with an amenable “non‐stick” interface is critical for observing true Brownian motion in LPTEM experiments. The field would benefitly greatly from additional study in systematically varying the electron beam dose rate to probe how the e‐beam results in these specific types of fractals.

## Conflicts of Interest

The authors declare no conflict of interest.

## Supporting information




**Supporting File**: smll72228‐sup‐0001‐SuppMat.docx.

**Supporting File**: smll72228‐sup‐0002‐VideoS1.avi.

**Supporting File**: smll72228‐sup‐0003‐VideoS2.avi.

**Supporting File**: smll72228‐sup‐0004‐VideoS3.avi.

**Supporting File**: smll72228‐sup‐0005‐VideoS4.avi.

**Supporting File**: smll72228‐sup‐0006‐VideoS5.avi.

## Data Availability

The data that support the findings of this study are available from the corresponding author upon reasonable request.

## References

[1] N. De Jonge and F. M. Ross , “Electron Microscopy of Specimens in Liquid,” Nature Nanotechnology 6, no. 11 (2011): 695–704, 10.1038/nnano.2011.161. 22020120

[2] C. M. Wang , H. G. Liao , and F. M. Ross , “Observation of Materials Processes in Liquids by Electron Microscopy,” MRS Bulletin 40, no. 1 (2015): 46–52, 10.1557/mrs.2014.283.

[3] J. Park , H. Park , P. Ercius , et al., “Direct Observation of Wet Biological Samples by Graphene Liquid Cell Transmission Electron Microscopy,” Nano Letters 15, no. 7 (2015): 4737–4744, 10.1021/acs.nanolett.5b01636. 26065925

[4] X. Chen and J. I. Wen , “In Situ Wet‐cell TEM Observation of Gold Nanoparticle Motion in an Aqueous Solution,” Nanoscale Research Letters 7 (2012): 1–6, 10.1186/1556-276X-7-598. 23107519 PMC3502471

[5] W. Kühlbrandt , “The Resolution Revolution,” Science 343 (2014): 1443–1444, 10.1126/science.1251652. 24675944

[6] R. S. Ruskin , Z. Yu , and N. Grigorieff , “Quantitative Characterization of Electron Detectors for Transmission Electron Microscopy,” Journal of Structural Biology 184, no. 3 (2013): 385–393, 10.1016/j.jsb.2013.10.016. 24189638 PMC3876735

[7] A. R. Faruqi , R. Henderson , and M. Pryddetch , “Direct Single Electron Detection With a CMOS Detector for Electron Microscopy,” Nuclear Instruments and Methods in Physics Research Section A: Accelerators, Spectrometers, Detectors and Associated Equipment 546 (2005): 170–175, 10.1016/j.nima.2005.03.023.

[8] N. H. Xuong , L. Jin , S. Kleinfelder , et al., “Future Directions for Camera Systems in Electron Microscopy,” Methods in Cell Biology 79 (2007): 721–739, 10.1016/S0091-679X(06)79028-8. 17327181

[9] G. Moldovan , X. Li , P. Wilshaw , and A. I. Kirkland , “Direct Electron Detectors for TEM,” presented at EMC 2008 14th European Microscopy Congress 1–5 September 2008, (Aachen, Germany) 2009, 1, 85–86, 10.1007/978-3-540-85156-1_43.

[10] G. Moldovan , X. Li , and A. Kirkland , “Can Direct Electron Detectors Outperform Phosphor‐CCD Systems for TEM?,” Journal of Physics: Conference Series 126 (2008): 012089, 10.1088/1742-6596/126/1/012089.

[11] E. A. Stach , D. Zakharov , R. D. Rivas , et al., “Exploiting a Direct Detection Camera for in‐situ Microscopy,” Microscopy and Microanalysis 19 (S2) (2013): 392–393, 10.1017/s1431927613003954.

[12] K. Gnanasekaran , H. Chang , P. J. M. Smeets , J. Korpanty , F. M. Geiger , and N. C. Gianneschi , “In Situ Ni2+Stain for Liposome Imaging by Liquid‐Cell Transmission Electron Microscopy,” Nano Letters 20, no. 6 (2020): 4292–4297, 10.1021/acs.nanolett.0c00898. 32453587

[13] J. M. Yuk , H. K. Seo , J. W. Choi , and J. Y. Lee , “Anisotropic Lithiation Onset in Silicon Nanoparticle Anode Revealed by in Situ Graphene Liquid Cell Electron Microscopy,” ACS Nano 8, no. 7 (2014): 7478–7485, 10.1021/nn502779n. 24980889

[14] J. P. Patterson , P. Abellan , M. S. Denny , et al., “Observing the Growth of Metal–Organic Frameworks by in Situ Liquid Cell Transmission Electron Microscopy,” Journal of the American Chemical Society 137, no. 23 (2015): 7322–7328, 10.1021/jacs.5b00817. 26053504

[15] L. R. Parent , K. Gnanasekaran , J. Korpanty , and N. C. Gianneschi , “100th Anniversary of Macromolecular Science Viewpoint: Polymeric Materials by in Situ Liquid‐Phase Transmission Electron Microscopy,” ACS Macro Letters 10, no. 1 (2021): 14, 10.1021/acsmacrolett.0c00595. 35548998

[16] U. M. Mirsaidov , H. Zheng , Y. Casana , and P. Matsudaira , “Imaging Protein Structure in Water at 2.7 nm Resolution by Transmission Electron Microscopy,” Biophysical Journal 102, no. 4 (2012): L15–L17, 10.1016/j.bpj.2012.01.009. 22385868 PMC3283772

[17] L. R. Parent , E. Bakalis , A. Ramírez‐Hernández , et al., “Directly Observing Micelle Fusion and Growth in Solution by Liquid‐Cell Transmission Electron Microscopy,” Journal of the American Chemical Society 139, no. 47 (2017): 17140–17151, 10.1021/jacs.7b09060. 29145727

[18] A. Rizvi , U. Patel , A. Ianiro , P. J. Hurst , J. G. Merham , and J. P. Patterson , “Nonionic Block Copolymer Coacervates,” Macromolecules 53, no. 14 (2020), https://doi.org/10.1021/ACS.MACROMOL.0C00979/SUPPL_FILE/MA0C00979_SI_004.MP4.

[19] M. A. Vratsanos and N. C. Gianneschi , “Direct Observation of Emulsion Morphology, Dynamics, and Demulsification,” ACS Nano 16 (2022): 7783–7793, 10.1021/ACSNANO.2C00199. 35302741 PMC9836053

[20] K. M. Vailonis , K. Gnanasekaran , X. B. Powers , N. C. Gianneschi , and D. M. Jenkins , “Elucidating the Growth of Metal–Organic Nanotubes Combining Isoreticular Synthesis With Liquid‐Cell Transmission Electron Microscopy,” Journal of the American Chemical Society 141, no. 26 (2019): 10177–10182, 10.1021/jacs.9b04586. 31244172

[21] M. Wang , A. C. Leff , Y. Li , and T. J. Woehl , “Visualizing Ligand‐Mediated Bimetallic Nanocrystal Formation Pathways With in Situ Liquid‐Phase Transmission Electron Microscopy Synthesis,” ACS Nano 15 (2021): 2578–2588, 10.1021/acsnano.0c07131. 33496576

[22] T. J. Woehl , J. E. Evans , I. Arslan , W. D. Ristenpart , and N. D. Browning , “Direct in Situ Determination of the Mechanisms Controlling Nanoparticle Nucleation and Growth,” ACS Nano 6, no. 10 (2012): 8599, 10.1021/NN303371Y. 22957797 PMC3482139

[23] B. L. Gilmore , S. P. Showalter , M. J. Dukes , et al., “Visualizing Viral Assemblies in a Nanoscale Biosphere,” Lab on A Chip 13, no. 2 (2012): 216–219, 10.1039/C2LC41008G. 23208001

[24] M. A. Touve , C. A. Figg , D. B. Wright , et al., “Polymerization‐Induced Self‐Assembly of Micelles Observed by Liquid Cell Transmission Electron Microscopy,” ACS Central Science 4, no. 5 (2018): 543–547, 10.1021/acscentsci.8b00148. 29806000 PMC5968509

[25] G. M. Scheutz , M. A. Touve , A. S. Carlini , et al., “Probing Thermoresponsive Polymerization‐Induced Self‐Assembly with Variable‐Temperature Liquid‐Cell Transmission Electron Microscopy,” Matter 4, no. 2 (2021): 722, 10.1016/j.matt.2020.11.017.

[26] A. Ianiro , H. Wu , M. M. J. van Rijt , et al., “Liquid–liquid Phase Separation During Amphiphilic Self‐Assembly,” Nature Chemistry 11, no. 4 (2019): 320–328, 10.1038/s41557-019-0210-4. 30778139

[27] A. Rizvi , J. T. Mulvey , and J. P. Patterson , “Observation of Liquid–Liquid‐Phase Separation and Vesicle Spreading during Supported Bilayer Formation via Liquid‐Phase Transmission Electron Microscopy,” Nano Letters 21, no. 24 (2021): 10325, 10.1021/acs.nanolett.1c03556. 34890211

[28] M. Nulati Yesibolati , K. I. Mortensen , H. Sun , A. Brostrøm , S. Tidemand‐Lichtenberg , and K. Mølhave , “Unhindered Brownian Motion of Individual Nanoparticles in Liquid‐Phase Scanning Transmission Electron Microscopy,” Nano Letters 20, (2020): 7108–7115, 10.1021/acs.nanolett.0c02352. 32678608

[29] A. Verch , M. Pfaff , and N. de Jonge , “Exceptionally Slow Movement of Gold Nanoparticles at a Solid/Liquid Interface Investigated by Scanning Transmission Electron Microscopy,” Langmuir 31, no. 25 (2015): 6956–6964, 10.1021/acs.langmuir.5b00150. 26050969

[30] E. R. White , M. Mecklenburg , B. Shevitski , S. B. Singer , and B. C. Regan , “Charged Nanoparticle Dynamics in Water Induced by Scanning Transmission Electron Microscopy,” Langmuir 28, no. 8 (2012): 3695, 10.1021/la2048486. 22320230 PMC3305795

[31] E. A. Ring and N. de Jonge , “Video‐Frequency Scanning Transmission Electron Microscopy of Moving Gold Nanoparticles in Liquid,” Micron 43, no. 11 (2012): 1078–1084, 10.1016/j.micron.2012.01.010. 22386765

[32] T. J. Woehl and T. Prozorov , “The Mechanisms for Nanoparticle Surface Diffusion and Chain Self‐Assembly Determined from Real‐Time Nanoscale Kinetics in Liquid,” The Journal of Physical Chemistry C 119, no. 36 (2015): 21261–21269, 10.1021/acs.jpcc.5b07164.

[33] E. Ring and N. De Jonge , “Hopping Diffusion of Gold Nanoparticles Observed with Liquid Cell TEM,” Microscopy and Microanalysis 22, no. 3 (2020): 750–751, 10.1017/S1431927616004608.

[34] S. W. Chee , Z. Baraissov , N. D. Loh , P. T. Matsudaira , and U. Mirsaidov , “Desorption‐Mediated Motion of Nanoparticles at the Liquid–Solid Interface,” The Journal of Physical Chemistry C 120, no. 36 (2016): 20462–20470, 10.1021/acs.jpcc.6b07983.

[35] M. N. Yesibolati , K. I. Mortensen , H. Sun , A. Brostrøm , S. Tidemand‐Lichtenberg , and K. Mølhave , “Unhindered Brownian Motion of Individual Nanoparticles in Liquid‐Phase Scanning Transmission Electron Microscopy,” Nano Letters 20, no. 10 (2020): 7108–7115, 10.1021/acs.nanolett.0c02352. 32678608

[36] L. R. Parent , E. Bakalis , M. Proetto , et al., “Tackling the Challenges of Dynamic Experiments Using Liquid‐Cell Transmission Electron Microscopy,” Accounts of Chemical Research 51, no. 1 (2018): 3–11, 10.1021/acs.accounts.7b00331. 29227618

[37] V. Jamali , C. Hargus , A. Ben‐Moshe , et al., “Anomalous Nanoparticle Surface Diffusion in LCTEM Is Revealed by Deep Learning‐Assisted Analysis,” Proceedings of the National Academy of Sciences 118, no. 10: 2017616118, 10.1073/pnas.2017616118. PMC7958372 33658362

[38] E. Bakalis , L. R. Parent , M. Vratsanos , C. Park , N. C. Gianneschi , and F. Zerbetto , “Complex Nanoparticle Diffusional Motion in Liquid‐Cell Transmission Electron Microscopy,” The Journal of Physical Chemistry C 124, no. 27 (2020): 14881–14890, 10.1021/acs.jpcc.0c03203. PMC8023318 33841603

[39] T. A. J. Welling , S. Sadighikia , K. Watanabe , et al., “Observation of Undamped 3D Brownian Motion of Nanoparticles Using Liquid‐Cell Scanning Transmission Electron Microscopy,” Particle & Particle Systems Characterization 37 (2020): 2000003, 10.1002/ppsc.202000003.

[40] S. W. Chee , U. Anand , G. Bisht , S. F. Tan , and U. Mirsaidov , “Direct Observations of the Rotation and Translation of Anisotropic Nanoparticles Adsorbed at a Liquid–Solid Interface,” Nano Letters 19, no. 5 (2019): 2871–2878, 10.1021/acs.nanolett.8b04962. 30932500

[41] N. de Jonge , L. Houben , R. E. Dunin‐Borkowski , and F. M. Ross , “Resolution and Aberration Correction in Liquid Cell Transmission Electron Microscopy,” Nature Reviews Materials 4, no. 1 (2018): 61–78, 10.1038/s41578-018-0071-2.

[42] J. Korpanty , L. R. Parent , and N. C. Gianneschi , “Enhancing and Mitigating Radiolytic Damage to Soft Matter in Aqueous Phase Liquid‐Cell Transmission Electron Microscopy in the Presence of Gold Nanoparticle Sensitizers or Isopropanol Scavengers,” Nano Letters 21 (2021): 1141–1149, 10.1021/acs.nanolett.0c04636. 33448858

[43] T. U. Dissanayake , M. Wang , and T. J. Woehl , “Revealing Reactions between the Electron Beam and Nanoparticle Capping Ligands with Correlative Fluorescence and Liquid‐Phase Electron Microscopy,” ACS Applied Materials & Interfaces 13 (2021): 37553, 10.1021/acsami.1c10957. 34338503

[44] T. J. Woehl and P. Abellan Baeza , “Defining the Radiation Chemistry during Liquid Cell Electron Microscopy to Enable Visualization of Nanomaterial Growth and Degradation Dynamics,” Journal of Microscopy 265 (2017): 135–147, 10.1111/jmi.12508. 27918613

[45] T. J. Woehl , K. L. Jungjohann , J. E. Evans , I. Arslan , W. D. Ristenpart , and N. D. Browning , “Experimental Procedures to Mitigate Electron Beam Induced Artifacts during in Situ Fluid Imaging of Nanomaterials,” Ultramicroscopy 127 (2013): 53–63, 10.1016/j.ultramic.2012.07.018. 22951261

[46] Q. Chen , J. M. Smith , J. Park , et al., “3D Motion of DNA‐Au Nanoconjugates in Graphene Liquid Cell Electron Microscopy,” Nano Letters 13, no. 9 (2013): 4556–4561, 10.1021/nl402694n. 23944844

[47] V. Jamali , C. Hargus , A. Ben‐Moshe , et al., “Anomalous Nanoparticle Surface Diusion in Liquid,” Cell TEM Is Revealed by Deep Learning‐Assisted Analysis (2020), ChemRxiv, 10.26434/CHEMRXIV.12894050.V2. PMC7958372 33658362

[48] K. M. Vailonis , K. Gnanasekaran , X. B. Powers , N. C. Gianneschi , and D. M. Jenkins , “Elucidating the Growth of Metal–Organic Nanotubes Combining Isoreticular Synthesis with Liquid‐Cell Transmission Electron Microscopy,” Journal of the American Chemical Society 141 (2019): 10177–10182, 10.1021/jacs.9b04586. 31244172

[49] J. P. Patterson , L. R. Parent , J. Cantlon , et al., “Picoliter Drop‐On‐Demand Dispensing for Multiplex Liquid Cell Transmission Electron Microscopy,” Microscopy and Microanalysis 22, no. 3 (2016): 507–514, 10.1017/S1431927616000659. 27135268 PMC5235328

[50] M. Wang , C. Park , and T. J. Woehl , “Quantifying the Nucleation and Growth Kinetics of Electron Beam Nanochemistry with Liquid Cell Scanning Transmission Electron Microscopy,” Chemistry of Materials 30, no. 21 (2018): 7727–7736, 10.1021/acs.chemmater.8b03050.

[51] A. Hutzler , B. Fritsch , M. P. M. Jank , et al., “In Situ Liquid Cell TEM Studies on Etching and Growth Mechanisms of Gold Nanoparticles at a Solid–Liquid–Gas Interface,” Advanced Materials Interfaces 6 (2019): 1901027, 10.1002/ADMI.201901027.

[52] H. Le Ferrand , M. Duchamp , B. Gabryelczyk , H. Cai , and A. Miserez , “Time‐Resolved Observations of Liquid‐Liquid Phase Separation at the Nanoscale Using in Situ Liquid Transmission Electron Microscopy,” J Am Chem Soc 141, no. 17 (2019): 7202–7210, 10.1021/JACS.9B03083/SUPPL_FILE/JA9B03083_SI_004.MPG. 30986043

[53] J. Y. Tinevez , N. Perry , J. Schindelin , et al., “TrackMate: An open and extensible platform for single‐particle tracking,” Methods 115 (2017): 80–90, 10.1016/J.YMETH.2016.09.016. 27713081

[54] D. Ershov , M. S. Phan , J. W. Pylvänäinen , et al., “TrackMate 7: Integrating state‐of‐the‐art segmentation algorithms into tracking pipelines,” Nature Methods 19 (7), 2022: 829–832, 10.1038/s41592-022-01507-1. 35654950

[55] P. Lançon , G. Batrouni , L. Lobry , and N. Ostrowsky , “Brownian Walker in a Confined Geometry Leading to a Space‐Dependent Diffusion Coefficient,” Physica A: Statistical Mechanics and its Applications 304 (2002): 65–76, 10.1016/S0378-4371(01)00510-6.

[56] Trackpy: Fast, Flexible Particle‐Tracking Toolkit — trackpy 0.5.0 documentation, http://soft‐matter.github.io/trackpy/v0.5.0/, (accessed May 2021).

[57] D. B. Allan , T. Caswell , N. C. Keim , C. M. van der Wel , and R. W. Verweij , Soft‐Matter/Trackpy: Trackpy v0.5.0 2021, 10.5281/ZENODO.4682814.

[58] C. Park , T. J. Woehl , J. E. Evans , and N. D. Browning , “Minimum Cost Multi‐Way Data Association for Optimizing Multitarget Tracking of Interacting Objects,” IEEE Transactions on Pattern Analysis and Machine Intelligence 2015, 37 (3), 611–624, 10.1109/TPAMI.2014.2346202. 26353265

[59] G. D. Vo and C. Park , “Robust Regression for Image Binarization under Heavy Noise and Nonuniform Background,” Pattern Recognition 2018, 81, 224–239, 10.1016/J.PATCOG.2018.04.005.

[60] L. P. Faucheux and A. J. Libchaber , “Confined Brownian Motion,” Physical Review E 1994, 49 (6), 5158–5163, 10.1103/PhysRevE.49.5158. 9961839

[61] E. Bakalis , S. Höfinger , A. Venturini , and F. Zerbetto , “Crossover of Two Power Laws in the Anomalous Diffusion of a Two Lipid Membrane,” The Journal of Chemical Physics 142 (21) 2015: 215102, 10.1063/1.4921891. 26049526

[62] A. Einsten , “The scientific work of Albert Einstein,” Annals of Nuclear Energy 1905, 7, 279–287, 10.1016/0306-4549(80)90076-6.

[63] C. Rodríguez‐Cruz , M. Molaei , A. Thirumalaiswamy , K. Feitosa , V. N. Manoharan , S. Sivarajan , D. H. Reich , R. A. Riggleman , and J. C. Crocker , “Experimental Observations of Fractal Landscape Dynamics in a Dense Emulsion,” Soft Matter 2023, 19 (35), 6805–6813, 10.1039/D3SM00852E. 37650227

[64] H. J. Hwang , R. A. Riggleman , and J. C. Crocker , “Understanding Soft Glassy Materials Using an Energy Landscape Approach,” Nature Materials 2016, 15 (9), 1031–1036, 10.1038/NMAT4663. 27322823

[65] F. D. A. A. Reis and V. R. Voller , “Universal Superdiffusion of Random Walks in Media with Embedded Fractal Networks of Low Diffusivity,” Physical Review E 2024, 110 (2), L022102, 10.1103/PhysRevE.110.L022102. 39295050

[66] S. Ratynskaia , K. Rypdal , C. Knapek , S. Khrapak , A. V. Milovanov , A. Ivlev , J. J. Rasmussen , and G. E. Morfill , “Superdiffusion and Viscoelastic Vortex Flows in a Two‐Dimensional Complex Plasma,” Physical Review Letters 2006, 96 (10), 105010, 10.1103/PhysRevLett.96.105010. 16605750

[67] B. Liu and J. Goree , “Superdiffusion in Two‐Dimensional Yukawa Liquids,” Physical Review E 2007, 75 (1), 016405, 10.1103/PhysRevE.75.016405. 17358266

[68] D. Stauffer , C. Schulze , and D. W. Heermann , “Superdiffusion in a Model for Diffusion in a Molecularly Crowded Environment,” Journal of Biological Physics 33 (4), (2008): 305–312, 10.1007/S10867-008-9075-2. PMC2646401 19669520

