# Supplementary material for: Liquid Phase TEM of Diffusing Emulsion Droplets
Source: Small. 2026 Jan 30;22(13):e12006. doi: 10.1002/smll.202512006 (PMC12954370; doi:10.1002/smll.202512006)
Supplement: Supplementary file 1 — Supporting File: smll72228‐sup‐0001‐SuppMat.docx. [file SMLL-22-e12006-s003.docx]

*Liquid Phase TEM of Diffusing Emulsion Droplets*

*Maria A. Vratsanos^a^_,_ Evangelos Bakalis^b^, Chiwoo Park^c^, Francesco Zerbetto^b^, and Nathan C. Gianneschi^a,d,e^**

*^a^ Department of Materials Science & Engineering, Northwestern University, Evanston, Illinois 60208, United States.*

*^b^ Department of Chemistry “G. Ciamician”, Universita di Bologna, Bologna, Italy.*

*^c^ Department of Industrial and Manufacturing Engineering, Florida State University, Tallahassee, Florida 32306, United States*

*^d^ International Institute for Nanotechnology, Chemistry of Life Processes Institute, Northwestern University, Evanston, Illinois 60208, United States*

*^c^ Department of Chemistry, Department of Biomedical Engineering, Department of Pharmacology, Northwestern University, Evanston, Illinois 60208, United States*

1. Materials
2. Sample Preparation
3. Liquid Cell Assembly
4. Microscope and Imaging Conditions
5. Resolution Estimation Calculations
6. Identification of Candidate Systems
7. Image Processing
8. Trajectory Extraction and Analysis
9. Supplemental Data Tables and Figures
10. Fractal Analysis
11. Fractal Dimension
12. List of Supplemental Videos
13. References
14. Materials

2, 2, 4-methylpentane (isooctane) was purchased from TCI America. Tetradecafluorohexane (perfluorohexane) was acquired from Alfa Aesar. Capstone FS-30 was purchased from ChemCruz. All other chemicals were purchased from Sigma Aldrich.

1. Sample Preparation

To prepare emulsions, surfactant was dissolved in the continuous phase before addition of the dispersed phase. The minor phase was dispersed via probe sonication or vortexing. Samples were prepared within an hour prior to imaging.

1. Liquid Cell Assembly

Liquid cells were assembled as previously reported.^1^ Briefly, emulsions were dropcast onto a prepared SiNx chip before sealing with another SiNx top chip and holder clamping mechanism. A Hummingbird Scientific Dual Flow Mixing holder was used, and the integrity of the sample cell was verified via an external pumping station prior to microscope insertion.

1. Microscope and Imaging Conditions

A JEM-ARM300F (JEOL Ltd., Tokyo, Japan) transmission electron microscope was used for *in situ* experiments at an operating voltage of at 300keV and current of 15µA (FEG source). Images were acquired with a Gatan 2k × 2k OneView-IS CMOS camera and a Gatan K3-IS direct electron detector (Gatan Inc., Pleasanton, CA, USA) via Gatan Digital Micrograph imaging software (Roper Technologies, Sarasota, FL, USA). Exposure durations ranged from 0.01s to 1s. Electron fluxes were measured by both the K3 and via the detected beam current, which has previously been calibrated via a Faraday Holder in conjunction with the respective apertures used. Video acquisition was done by either the *in situ* camera functionalities or by screen recording with Camtasia Studio 2018 (TechSmithCorporation, USA). Frame rates for each data set are as indicated in figure captions.

1. Resolution Estimation Calculations

In the bulk state, a spherical particle in a fluid having a low Reynolds number will exhibit a diffusivity given by the Stokes-Einstein formula (Equation 1), which is dependent on particle size (R), solvent viscosity (η), and temperature (T).^2^

|  | $D=\frac{k_{B}T}{4\pi\eta R}$ | **Equation S1** |
| --- | --- | --- |

This relation is for two dimensions so long as the particle is sufficiently far from boundaries and other particles, making it possible to ignore the drag imposed by any walls or interparticle forces. However, when a particle exists in a more confined environment, such as between two parallel plates, it is not sufficiently free to explore that space, leading to deviations from the above expression.^3–5^ Rather, the influence of drag near the walls, while negligible on the bulk scale, significantly hinders the particle’s progress and slows its motion.^6^ The extent of this confinement with respect to the size of the particle will determine the degree of reduction in diffusivity. Aforementioned efforts to image this motion *in situ* have repeatedly resulted in anomalous behavior and calculated diffusivities that are two to nine orders of magnitude below expected values given by Equation **S1**.^7^

Given that the SiNx liquid cell geometry can be approximated as two parallel plates, we can draw from literature exploring particle motion when confined in one dimension, such as the work of Faucheux *et al*., wherein the motion of micron-scale polystyrene particles between two glass slides was recorded and quantified.^8^ These experiments showed that the reduction in diffusivity was directly related to a parameter γ, defined as a dimensionless variable relating the particle’s average z position to its size (Equation S2). We have reproduced their calculations here such that they may be adapted for our approximation of the *in situ* environment.^8^

|  | $\gamma=\frac{h-r}{r}$ | **Equation S2** |
| --- | --- | --- |

Here, r is defined as the particle radius and h is calculated from the Boltzmann density profile for particles of a given size and density in a fluid (**Equation S3**).

|  | $P_{B}\left( z \right)=\left( \frac{1}{L} \right)(\frac{e^{-\frac{z}{L}}}{e^{-\frac{r}{L}}-e^{\frac{\left( r-t \right)}{L}}})$ | **Equation S3** |
| --- | --- | --- |

t is the thickness of the liquid layer, and L is the characteristic Boltzmann length scale (**Equation S4**)

|  | $L=\frac{k_{B}T}{\frac{4}{3}\pi r^{3}g(\rho-\rho_{o})}$ | **Equation S4** |
| --- | --- | --- |

We can then define h as the following:

|  | $h=\int_{r}^{t-r} zP_{B}\left( z \right)dz=\frac{e^{-\frac{r}{L}}\left[ rL+L^{2} \right]-e^{\frac{r-t}{L}}[\left( t-r \right)L+L^{2}]}{L(e^{-\frac{r}{L}}-e^{\frac{r-t}{L}})}$ | **Equation S5** |
| --- | --- | --- |

Empirically, the reduction in diffusivity as a function of γ is shown in Faucheux et. al’s work, which can be approximated as Equation 6.^8^

|  | $\frac{D}{D_{o}}=0.0002\gamma^{3}-0.008\gamma^{2}+0.1069\gamma+0.3333$ | **Equation S6** |
| --- | --- | --- |

The theoretical considerations necessary to calculate diffusivity in bulk or constrained environments have been previously described (SI, Section V where Equation S1 provides the unconstrained bulk diffusivity).^53,54^ Briefly, by entering the geometrical constraints of the liquid cell and the solvent and particle properties, we can obtain (*via* Equations S2-S6) an estimate of what effective diffusivity should be and can approximate a particle’s mean square displacement, which can tell us what the minimum image capture rate should be to successfully image a given displacement (**Figure 1**). Here, for a typical liquid cell, we assume a uniform liquid thickness of 500 nm. Considering a range of nanoparticles with typical sizes (diameters ranging from 50 to 450 nm), we can see how their diffusivity in the liquid cell will vary from the bulk, and, intuitively, that this effect becomes more pronounced as the particle size approaches the length scale of the constrained environment (**Figure 1A**).

Using the same size range of particles, it is also possible to calculate the frame rate at which images would need to be captured to record displacements of a given magnitude (**Figure 1B**). Here, capturable displacements range linearly from 10 nm to 1 µm and the reduced diffusivity coefficient is used to calculate the time step required for the particle to travel that distance. The inverse of this time step is taken to give the required frame rate. Different particle tracking algorithms rely on different mechanisms of interpolation, or guessing the pathway from point A to point B for a given particle. Most visual tracking methods rely on linking algorithms, which search for a predefined radius around an identified particle. If no particles are found within that radius for a set number of subsequent frames, the particle trajectory is ended. This parameter of linking radius can significantly impact findings and conclusions in a data set with mean adjacent particles, as it can be nearly impossible to distinguish them from one another in experimental data. Thus, linking accuracy is inversely proportional to linking radius, as expanding the search area inherently increases the odds of misidentifying particles. However, as seen in **Figure 1B**, shrinking the detected mean square displacement quickly outpaces the capabilities of the camera, indicating the need to find an optimum between tracking accuracy and image acquisition. The superimposed box indicates the ranges of droplet sizes and imaging parameters considered in the experimental portion of this paper.

The above calculations may also be used to identify optimal candidate systems for studying nanoscale dynamics *in situ* – particles of various sizes and densities may be put into solvents of varying viscosities and thicknesses, and the same frame rate measurement may be done to estimate whether it is feasible to capture the unencumbered dynamics of that particular system with the available hardware (*i.e*., camera). For instance, a common strategy for observing dynamics is to suspend nanoparticles in a solvent mixed with glycerol, as its high viscosity rapidly slows motion (**Figure S1**).

Another critical consideration here is the relationship between frame rate and image resolution. Camera frame rate is inversely proportional to signal acquisition, resulting in a conundrum of image processing – the microscopist is forced to try to extract the same information about particles with less and less information. While there are many useful tools for this purpose, such as image segmentation and binarization algorithms, denoising functions, and machine learning, these are only able to help to a degree. That is, it is not possible to enhance contrast that is not there.^37,55^ The resolution of the microscope is inversely proportional to electron fluence and is additionally a function of sample and microscope conditions (**Figure 1C**).^56^ Here, we have adapted the calculations of deJonge^56^ to our typical sample parameters and electron fluences (see SI for details). For the organic materials used here, we typically consider low dose to be an electron flux of 0.2 e^-^/Å^2^s, to avoid damage to the sample. However, at the frame rates established in **Figure 1B**, this translates to a fluence per image of <0.02e^-^/Å^2^. At these rates, we cannot hope to discern objects less than 50 nm in diameter. As such, we have shifted out of necessity to larger, but more mobile emulsion droplets and have utilized advanced low contrast feature detection algorithms.^57^ The algorithm adopts a robust statistics approach to identify and subtract the image background due to uneven illumination and background material, and each of the remaining foreground images are fitted by an ellipse model to identify elliptical boundaries of particles.

In addition to the considerations outlined above, we also considered the strategy of increasing solvent viscosity to ease visualization of particle motion by slowing the particles, such as by the addition of glycerol.^9–11^ As such, we have calculated the imaging rates necessary for particles in solvents of viscosity ranging from that of pure water to that of pure glycerol (**Figure S**2).


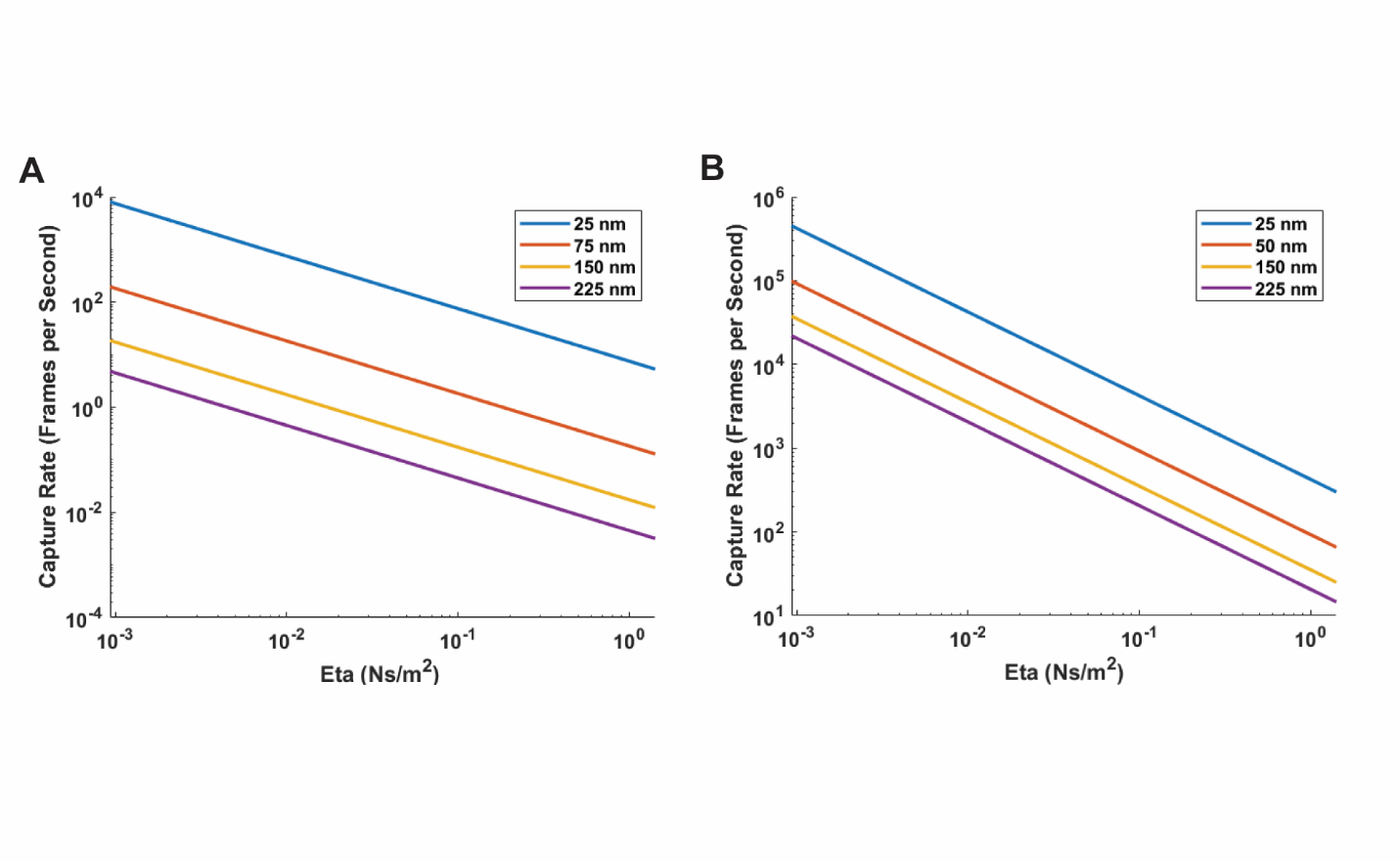


**Figure S**2 Capture rate as solvent viscosity varies from water to glycerol. (A) considers a displacement proportional to droplet size (3*r), and (B) considers a constant displacement of 10 nm

In order to consider the limitations of signal as it relates to resolution, we utilized the work of deJonge, whose source code was provided in the supplemental information.^12^ Such code was modified to match the typical sample parameters of the materials used in this work (window and liquid thickness, solvent density, *etc*.).

1. Identification of Candidate Systems

Significant efforts were made to observe polymeric nanoparticles in aqueous solutions (predominantly, polystyrene nanoparticles of various sizes). However, the low density differential between the particles of interest and the solvent made these impossible to observe on the desired temporal resolution.

1. Image Processing

High bandpass filters were used to reduce the noise inherent to low dose, high framerate imaging and to mitigate the gradient of contrast inherent to variable liquid thickness.

1. Trajectory Extraction and Analysis

In videos with sufficient contrast, manual tracking was done in ImageJ using open source plugins^13,14^. Once acquired, the trajectories were used to calculate mean square displacement in MATLAB. MSD was plotted as a function of lag time on log-log axes, and a power law fitting was applied (of the form *Ax^n^ +B*, where n indicates the nature of the motion, and ¼ * A yields the diffusivity coefficient, D).^15^

1. Supplemental Data Tables

Some trajectories were not of sufficient length for ADOMA analysis, and thus were not included in **Figure 3**. We have compiled the tabulated data for those shorter trajectories below (**Table S1**). Here, the number of time points indicates the number of video frames over which the droplet in question was tracked. Droplet size was measured by the tracking algorithm. Stokes-Einstein and reduced diffusivities were calculated via Equations 1 and 6, respectively, in the main text. MSD coefficient reflects the coefficient of the power law fitting, and MSD diffusivity is this coefficient divided by 4. MSD Exponent is the exponent value from the power law fitting. Average values for each are listed at the bottom of the table.

|  |  |  |  |  |  |  |  |  |
| --- | --- | --- | --- | --- | --- | --- | --- | --- |
|  |  |  |  |  |  |  |  |  |
|  |  |  |  |  |  |  |  |  |
|  |  |  |  |  |  |  |  |  |
|  |  |  |  |  |  |  |  |  |
|  |  |  |  |  |  |  |  |  |
|  |  |  |  |  |  |  |  |  |
|  |  |  |  |  |  |  |  |  |
|  |  |  |  |  |  |  |  |  |
|  |  |  |  |  |  |  |  |  |
|  |  |  |  |  |  |  |  |  |
|  |  |  |  |  |  |  |  |  |
|  |  |  |  |  |  |  |  |  |

1. Fractal Analysis

Details of the method analysis have been given elsewhere.^11^ The key element of the analysis is the scaling of the metric <||ΔX_i_||^q^>, where ||ΔX_i_|| is the Euclidean distance of the increments ΔX_i_, with X_i_ being the elements of the recorded time series along *x-y* axes. The parameter *q* expresses the order of the moment; in this work the parameter *q* is varied in the range [0.25, 4] with a step of 0.25. For the lateral motion, we create the sequence $l_{i}=\sqrt{x_{i}^{2}+y_{i}^{2}}$. We assume that the recording time series, at least for time lags much smaller than the total length, pose a kind of self-similarity, zoomed in or out of the time series reveal the same patterns scaled by a certain amount, and accordingly we expect Equation S7 to be satisfied.^16^

<||ΔX_i_||^q^> ≈τ^z(q)^ **Equation S7**

In **Equation S7**, τ is the time lag and *z(q)* is the structure function – its form provides insights on the stochastic mechanisms affecting the motion. The value of *z(q)* for q=1 corresponds to the Hurst exponent, and for q=2 it returns the scaling of the MSD, if and only if a single origin of noise is present, *z(q)*=hq, (**Table S2**). Instead, if more noise sources contribute and drive the motion then *z(q)* departs from linearity and its convex shape is signature of multiplicative effects. Two special forms of *z(q)* are *z(q)=hq-c(q^2^-q)* for log-normal distribution, and *z(q)*=*hq-cqlog(q)* for log-Cauchy distribution. Both are special cases of $z\left( q \right)=hq-\frac{c}{a-1}(q^{a}-q)$ for *a*=2 and 1 respectively. The exponent *a* takes values in the range (0,2], *a*-stable distribution.

**Table S2** Details of Structure Functions

| Trajectory | | Number of timepoints | | Structure Function  z(q) | | x-axis | y-axis | Lateral |
| --- | --- | --- | --- | --- | --- | --- | --- | --- |
| **Perfluorohexane** | | | | | | | | |
| PFH_1 | | 2052 | | z(q)=hq-c(q^2^-q) | h = 0.538 +/- 0.001  c= 0.016 +/- 0.001 | | h = 0.427 +/- 0.001  c = 0.012 +/- 0.001 | h = 0.470 +/- 0.001  c= 0.013 +/- 0.001 |
| PFH_2 | | 232 | | z(q)=hq-c(q^2^-q)  z(q)=hq | h =0.887 +/-0.003  c= 0.107 +/- 0.001 | | h= 0.640 +/- 0.008 | h= 0.817 +/- 0.008  c= 0.089 +/- 0.004 |
| **Isooctane** | | | | | | | | |
| Iso_1 | 248 | | z(q)=hq-c(q^2^-q)  z(q)=hq | | h= 0.359+/- 0.001  c= 0.022 +/- 0.001 | | h = 0.508 +/- 0.003  c = 0.067 +/- 0.001 | h = 0.385 +/- 0.001 |
| Iso_2 | 104 | | z(q)=hq-c(q^2^-q)  z(q)=hq | | h= 0.336+/-0.002 | | h= 0.371+/- 0.002  c= 0.027 +/- 0.001 | h= 0.350 +/- 0.001 |
| Iso_3 | 260 | | z(q)=hq  z(q)=hq-(c/(a-1))(x^a^-x) | | h= 0.475+/-0.002 | | h= 0.488 +/- 0.001  c= 0.109 +/- 0.002  a= 1.327 +/- 0.020 | h= 0.51 +/- 0.001 |
| Iso_4 | 140 | | z(q)=hq-c(q^2^-q) | | h= 0.511+/- 0.004  c= 0.079+/- 0.002 | | h= 0.502 +/- 0.002  c= 0.045 +/- 0.001 | h= 0.494 +/- 0.004  c= 0.079 +/- 0.002 |
| Iso_5 | 605 | | z(q)=hq-c(q^2^-q)  z(q)=hq | | h= 0.345+/- 0.004  c= 0.030+/- 0.002 | | h= 0.248 +/- 0.004  c= 0.037 +/- 0.002 | h= 0.368 +/- 0.001 |

**Table S3 Hurst Exponents** $H=z(q=1)$, for a truly Brownian motion H=1/2, and the scaling of the MSD and/or variance, $<\Delta x^{2}>\sim t^{n}$, is n=2H. It should be noted that the scaling of the second moment, n is equal to $z(q=2)$.

| Trajectory | Number of timepoints | x-axis | y-axis | Lateral |
| --- | --- | --- | --- | --- |
| **Perfluorohexane** | | | | |
| PFH_1 | 2052 | z(q=1)=0.54  z(q=2) = 1.04  var ~ 0.98 | z(q=1)=0.43  z(q=2) = 0.83  var ~ 0.79 | z(q=1)=0.47  z(q=2) = 0.92  var ~ 0.89 |
| PFH_2 | 232 | z(q=1)=0.88  z(q=2) = 1.57  var ~ 1.31 | z(q=1)=0.73  z(q=2) = 1.36  var ~ 1.15 | z(q=1)=0.78  z(q=2) = 1.48  var ~ 1.41 |
| **Isooctane** | | | | |
| Iso_1 | 248 | z(q=1)=0.36  z(q=2) = 0.67  var ~ 0.57 | z(q=1)=0.52  z(q=2) = 0.88  var ~ 0.61 | z(q=1)=0.37  z(q=2) = 0.76  var ~ 0.79 |
| Iso_2 | 104 | z(q=1)=0.36  z(q=2) = 0.69  var ~ 0.63 | z(q=1)=0.38  z(q=2) = 1.04  var ~ 0.56 | z(q=1)=0.33  z(q=2) = 0.69  var ~ 0.72 |
| Iso_3 | 260 | z(q=1)=0.50  z(q=2) = 0.96  var ~ 0.90 | z(q=1)=0.49  z(q=2) = 0.81  var ~ 0.60 | z(q=1)=0.49  z(q=2) = 1.00  var ~ 1.04 |
| Iso_4 | 140 | z(q=1)=0.52  z(q=2) = 0.96  var ~ 0.65 | z(q=1)=0.49  z(q=2) = 0.92  var ~ 0.84 | z(q=1)=0.51  z(q=2) = 0.83  var ~ 0.62 |
| Iso_5 | 605 | z(q=1)=0.36  z(q=2) = 0.63  var ~ 0.51 | z(q=1)=0.27  z(q=2) = 0.42  var ~ 0.30 | z(q=1)=0.37  z(q=2) = 0.74  var ~ 0.74 |

Following the same path of analysis applied for droplet PFH_1 and discussed in the main text we illustrate in **Figure S2** the main findings for the droplet PFH_2. We observe that: i) NVAF retains a memory of the order of 10/15 steps for x-/y-axes, **Figure S2D**, ii) excess kurtosis shows a Gaussian type distribution for motion in y-axis and of a non-Gaussian type for x-axis and lateral motion, **Figure S2C**, iii) the probability distribution for waiting times is not conclusive for cut-off distance of twice the standard deviation and we reset it at one standard deviation, and iv) the motion is super-diffusive, (**Table S3**). Observe that, with the exception of a very small number of steps that are roughly equal to half the radius, the lenghts of the increments are very small in relation to the droplet radius, **Figure S2E**. In addition, the calculated fractal dimension (*d_f_* = 1.17), see **Figure 6** in main text and **Table S5**, underlines the existence of few available paths, a picture consistent with the trajectory of the droplet on the FoV(**Figure S2A)**. Given that the waiting times to overcome a distance of one standard deviation correspond to an equivalent barrier of about 16.5 kcal/mol, this is not much different than the barrier of 17 kcal/mol found for the PFH_1 droplet. Such a barrier common in both droplets can be imposed by the e− beam rearranging the energy landscape. The only thing left is the surface tension resulting from the extreme compression of the droplet within the cell. These tensions drive the motion from energy minimum to energy minimum since the landscape is constantly reorganised and leads to super diffusive motion, a result in agreement with previous studies.^12,13^ There are traces of the highly conjugated motion in the x- and y-axes in the corresponding structure functions. The combined lateral motion has a linear shape, but motion along the y-axis has a linear shape and is convex along the x-axis. This indicates that despite the average distance between consecutive points increases linearly as lag time increases for motion along the y-axis, the mean intermittency—the parameter c of the structure function—imposed by the x-axis drives the overall motion; see the values of the structure function in **Table S2** as well as the values of z for q = 1, 2, and the scaling exponent of the variance in **Table S3**.


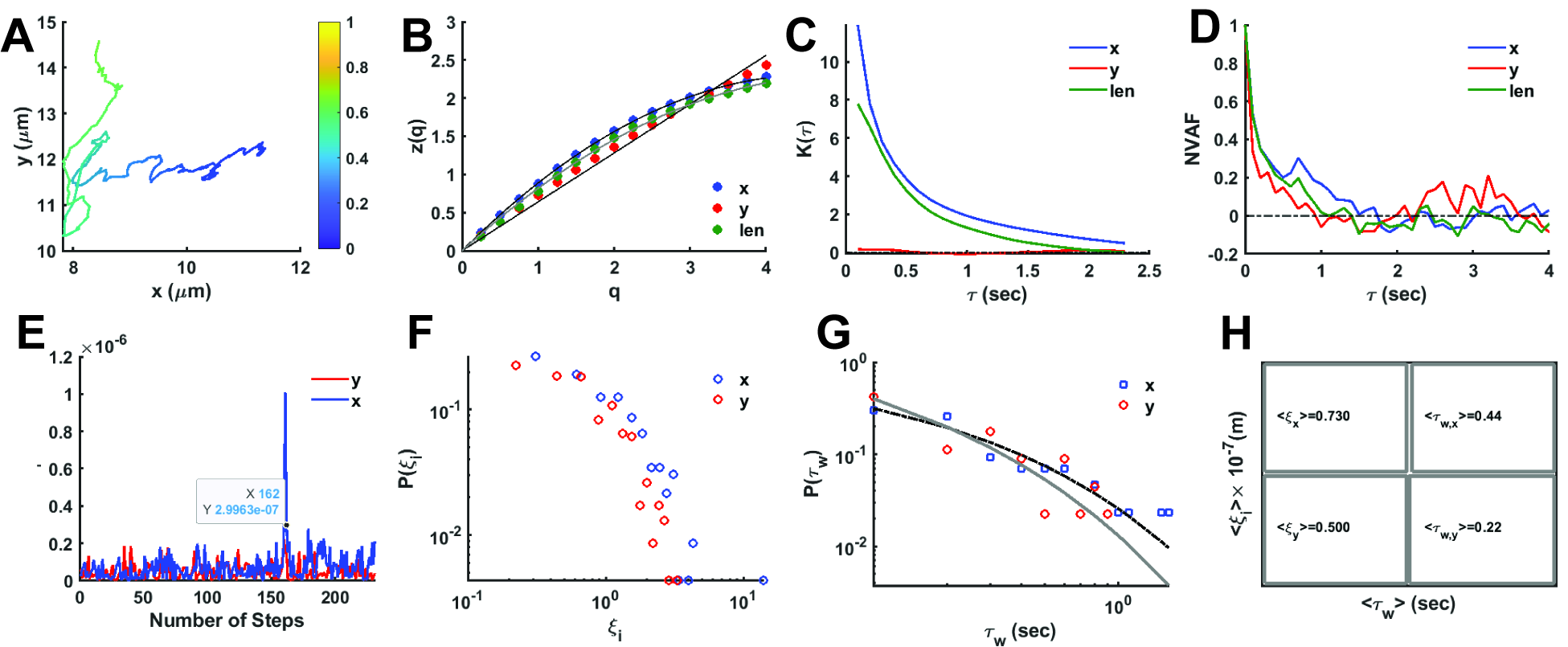


***Figure S2*** *ADOMA results for PFH_2 (A) Motion trajectory, (B) structure functions, (C) excess kurtosis, (D) normalized velocity autocorrelation functions, (E) movement increments along x- and y-axes, (F) probability distribution of ξ_i_=||ΔX_i_||/<||ΔX_i_||>, (G) probability distribution of waiting times, whereas the value ξ_i_=2s (s is the standard deviation) has been set as cut-off distance, g) probability distribution of waiting times, and (H) mean of the length take along x- and y- axes and the mean of the waiting time, for the particle PFH_2 are displayed.*


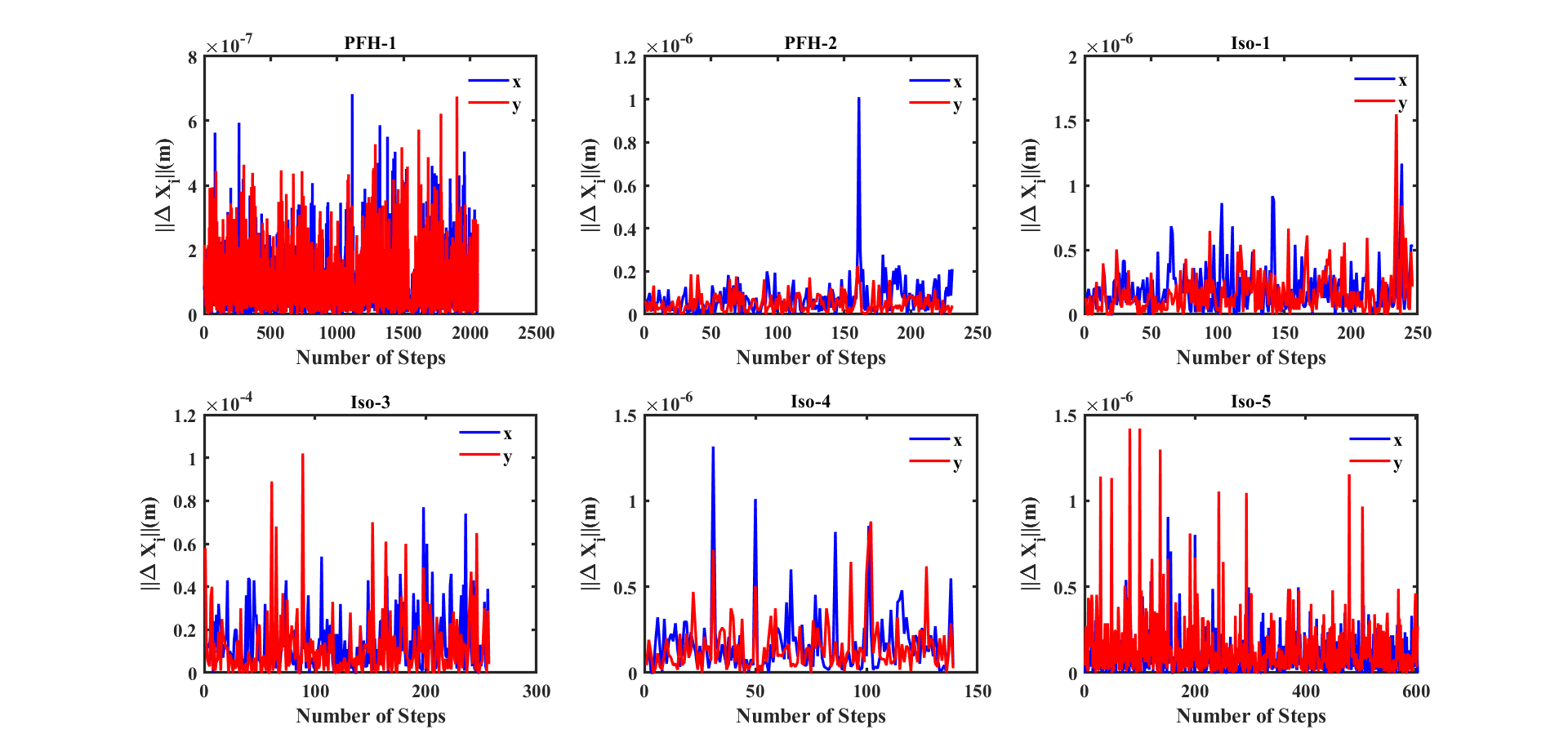


***Figure S3*** *Increments ||ΔX_i_|| along x- and y-axes (blue and red respectively). It is worth noting that increments are much larger for isooctane droplets.*

**Table S4** Parameters of best fittings for distribution of waiting times for PFH droplets. Log normal $P\left( \tau\right)=\frac{a}{\tau b\sqrt{2\pi}}e^{-\frac{(\log\left( \tau\right)-c)^{2}}{2b^{2}}}$ works well for PFH_1, and a power law truncated by a special stretched exponential $P\left( \tau\right)=\tau^{-d-1}e^{-f\sqrt{\tau}}$ for droplet PFH_2.

| PFH_1 | *axis* | *a* | *b* | *c* |
| --- | --- | --- | --- | --- |
|  | *x* | 0.112±0.007 | 1.222±0.081 | -0.866±0.079 |
|  | *y* | 0.120±0.010 | 1.374±0.132 | -0.964±0.105 |
| PFH_2 | *axis* | *d* | *f* |  |
|  | *x* | -0.996±0.058 | 3.671±0.255 |  |
|  | *y* | -0.799±0.100 | 4.345±0.540 |  |

Section XI. Fractal Dimension

Fractals are natural objects that cannot be described in terms of simple primitives. They describe never-ending patterns, which are clones of the same pattern regardless of the magnification, a property also known as self-similarity. Mandelbrot, in his seminal work “How long is the coast of Britain?”^14^ coined the term “fractals” and made a significant contribution pointing to a non-integer geometry.^15^ Fractal dimension, *d_f_* , or Hausdorff dimension, is the descriptor that provides the degree of self- similarity in the complex geometry of a fractal structure. It is defined as *L(r) ∼ r^df^* , where *L* is the length of the fractal for a given resolution of the measuring device r. Calculating the fractal dimension is not an easy task, and several methods have been proposed to deal with that.^16^ The box-counting method is a classical approach to calculating fractal dimension. In D-dimensional space (D=1,2,3), it begins with mapping the fractal and views the entire figure as a square whose side is a power of two. Then, we split the initial square into four squares, each one with side the half of the initial one. We repeat the process to reach the maximum resolution of the map. Let’s assume that in each step, the side of a square is ϵ and the number of squares contributing to the length is N. The theoretical box- counting dimension is given as $d_{f}= \lim_{\in\to0} \frac{log(N)}{log(\frac{1}{\epsilon})}$. For real life data, the limit of zero for ϵ cannot be approached. Instead, we use the following equation:

$N\left( \epsilon\right)=A\epsilon^{-df}$ **Equation S9**

and we calculate the fractal dimension as the absolute value of the slope of the linear regression in log_2_-log_2_ space of **Equation S9**. We run a home-made code whose work flow follows the steps:

1. Find the minimum and maximum values in both x- and y-axes and define the range *r_i_ = max_i_ − min_i_*, *i = x, y*, with *range = max(r_x_, r*y*)*.
2. Find the minimum increment in *Δ_min_ =min(Δ_x_, Δ_y_*)
3. Define the maximum square where the entire information will be stored, with side $s=\frac{range}{\Delta_{min}}$. For computational memory reasons, we fix the maximum square with 1024×1024, so if *s* > 1024, we impose a proper truncation on the minimum increment, which is the minimum of the half standard deviation of the increments along the x- and y-axes.
4. Convert the spatial information of the recorded coordinates to binary information: 1 if the cell (square) contains part of the walk and 0 otherwise.
5. Divide the entire square according to squares with length sides ϵ^2n^, n = 0, 1, 2, , ,.
6. At each run, check if a square contains a value of 1, and if yes, then take it into account.
7. Plot in log_2_-log_2_ the number of squares versus the length side of them. The absolute value of the slope is the fractal dimension, *d_f_* .

**Table S5** Symbols used in this table: NVAF= Normalized Velocity Autocorrelation Function,

$K\left( \tau\right)=\frac{(<X^{4}>)}{3<X^{2}>^{2}}-1$ provides the Gaussian character of the distribution when it is zero. The fractal dimension, d_f_ of the walk on the field of view. MF stands for multifractal motion, and CD for confined diffusion.

| Trajectory | Axis | Shape of Structure Function | NVAF | *K*(τ) | d_f_ | Mechanism |
| --- | --- | --- | --- | --- | --- | --- |
| PFH_1 | x | Convex | >0 | <0 |  | MF |
|  | y | Convex | >0 | ≠0 |  | MF |
|  | x-y |  |  |  | 1.42 | RWF |
|  | Lateral | Convex | >0 | <0 |  | MF |
| PFH_2 | x | Convex | >0 | >0 |  | MF |
|  | y | Linear | >0 | =0 |  | CD |
|  | x-y |  |  |  | 1.17 | RWF |
|  | Lateral | Convex | >0 | >0 |  | MF |
| Iso_1 | x | Convex | <0 | <0 |  | MF |
|  | y | Convex | <0 | <0 |  | MF |
|  | x-y |  |  |  | 1.31 | RWF |
|  | Lateral | Linear | <0 | >0 |  | CD |
| Iso_2 | x | Linear | <0 | <0 |  | CD |
|  | y | Convex | <0 | <<0 |  | MF |
|  | x-y |  |  |  | 1.32 | RWF |
|  | Lateral | Linear | <0 | ~0 |  | CD |
| Iso_3 | x | Linear | <0 | ~0 |  | CD |
|  | y | Convex | <0 | <0 |  | MF |
|  | x-y |  |  |  | 1.20 | RWF |
|  | Lateral | Linear | <0 | ~0 |  | CD |
| Iso_4 | x | Convex | Delta type | <0 |  | MF |
|  | y | Convex | <0 | <0 |  | MF |
|  | x-y |  |  |  | 1.18 | RWF |
|  | Lateral | Convex | Delta type | <0 |  | MF |
| Iso_5 | x | Convex | <0 | ~0 |  | MF |
|  | y | Convex | <0 | ~0 |  | MF |
|  | x-y |  |  |  | 1.44 | RWF |
|  | Lateral | Convex | <0 | =0 |  | MF |

1. List of Supplemental Videos

Video S1: Videographic data of isooctane droplets stabilized by AOT in water, from which trajectories of droplets Iso_1 to Iso_4 and Iso_6 to Iso_9 were extracted

Video S2: Videographic data of trajectory of droplet PFH_1 in aqueous liquid cell

Video S3: Videographic data showing trajectory of droplet PFH_2

Video S4: Videographic data of isooctane droplets from which Iso_10 and Iso_11 trajectories were extracted

VideoS5: Videographic data of isooctane droplets from which trajectories of droplets Iso_12 through Iso_16 were extracted

1. References

(1) Vratsanos, M. A.; Gianneschi, N. C. Direct Observation of Emulsion Morphology, Dynamics, and Demulsification. *ACS Nano* **2022**, acsnano.2c00199. https://doi.org/10.1021/ACSNANO.2C00199.

(2) Einsten, A. On the Motion of Small Particles Suspended in Liquids at Rest Required by the Molecular-Kinetic Theory of Heat. *Ann Phys* **1905**, *17*, 549–560. https://doi.org/10.1016/0306-4549(80)90076-6.

(3) Burada, P. S.; Hänggi, P.; Marchesoni, F.; Schmid, G.; Talkner, P. *Diffusion in Confined Geometries*; 2009; Vol. 10. https://doi.org/10.1002/cphc.200800526.

(4) Bezrukov, S. M.; Schimansky-Geier, L.; Schmid, G. Brownian Motion in Confined Geometries. *Eur. Phys. J. Special Topics* **2014**, *223*, 3021–3025. https://doi.org/10.1140/epjst/e2014-02316-6.

(5) Borodin, A. N.; Salminen, P. *Handbook of Brownian Motion — Facts and Formulae*; Birkhäuser Basel, 1996. https://doi.org/10.1007/978-3-0348-7652-0.

(6) Faucheux, L. P.; Libchaber, A. J. Confined Brownian Motion. *Phys Rev E* **1994**, *49* (6), 5158–5163. https://doi.org/10.1103/PhysRevE.49.5158.

(7) Nulati Yesibolati, M.; Mortensen, K. I.; Sun, H.; Brostrøm, A.; Tidemand-Lichtenberg, S.; Mølhave, K. Unhindered Brownian Motion of Individual Nanoparticles in Liquid Phase Scanning Transmission Electron Microscopy. **2020**. https://doi.org/10.1021/acs.nanolett.0c02352.

(8) Faucheux, L. P.; Libchaber, A. J. Confined Brownian Motion. *Phys Rev E* **1994**, *49* (6), 5158–5163. https://doi.org/10.1103/PhysRevE.49.5158.

(9) Verch, A.; Pfaff, M.; de Jonge, N. Exceptionally Slow Movement of Gold Nanoparticles at a Solid/Liquid Interface Investigated by Scanning Transmission Electron Microscopy. *Langmuir* **2015**, *31* (25), 6956–6964. https://doi.org/10.1021/acs.langmuir.5b00150.

(10) Yesibolati, M. N.; Mortensen, K. I.; Sun, H.; Brostrøm, A.; Tidemand-Lichtenberg, S.; Mølhave, K. Unhindered Brownian Motion of Individual Nanoparticles in Liquid-Phase Scanning Transmission Electron Microscopy. *Nano Lett* **2020**, *20* (10), 7108–7115. https://doi.org/10.1021/acs.nanolett.0c02352.

(11) Ring, E. A.; de Jonge, N. Video-Frequency Scanning Transmission Electron Microscopy of Moving Gold Nanoparticles in Liquid. *Micron* **2012**, *43* (11), 1078–1084. https://doi.org/10.1016/j.micron.2012.01.010.

(12) de Jonge, N. Theory of the Spatial Resolution of (Scanning) Transmission Electron Microscopy in Liquid Water or Ice Layers. *Ultramicroscopy* **2018**, *187*, 113–125. https://doi.org/10.1016/J.ULTRAMIC.2018.01.007.

(13) Tinevez, J. Y.; Perry, N.; Schindelin, J.; Hoopes, G. M.; Reynolds, G. D.; Laplantine, E.; Bednarek, S. Y.; Shorte, S. L.; Eliceiri, K. W. TrackMate: An Open and Extensible Platform for Single-Particle Tracking. *Methods* **2017**, *115*, 80–90. https://doi.org/10.1016/J.YMETH.2016.09.016.

(14) Ershov, D.; Phan, M. S.; Pylvänäinen, J. W.; Rigaud, S. U.; Le Blanc, L.; Charles-Orszag, A.; Conway, J. R. W.; Laine, R. F.; Roy, N. H.; Bonazzi, D.; Duménil, G.; Jacquemet, G.; Tinevez, J. Y. TrackMate 7: Integrating State-of-the-Art Segmentation Algorithms into Tracking Pipelines. *Nature Methods 2022 19:7* **2022**, *19* (7), 829–832. https://doi.org/10.1038/s41592-022-01507-1.

(15) Allan, D. B.; Caswell, T.; Keim, N. C.; van der Wel, C. M.; Verweij, R. W. Soft-Matter/Trackpy: Trackpy v0.5.0. **2021**. https://doi.org/10.5281/ZENODO.4682814.

(16) Bakalis, E.; Höfinger, S.; Venturini, A.; Zerbetto, F. Crossover of Two Power Laws in the Anomalous Diffusion of a Two Lipid Membrane. *Journal of Chemical Physics* **2015**, *142* (21). https://doi.org/10.1063/1.4921891.

(17) Meroz, Y.; Sokolov, I. M.; Klafter, J. Test for Determining a Subdiffusive Model in Ergodic Systems from Single Trajectories. *Phys Rev Lett* **2013**, *110* (9). https://doi.org/10.1103/PHYSREVLETT.110.090601.

(18) Dasgupta, R.; Ballabh, T. K.; Tarafdar, S. Scaling  Exponents for Random Walks on Sierpinski Carpets and Number of Distinct Sites  Visited:  A New Algorithm for Infinite Fractal Lattices. *J Phys A Math Gen* **1999**, *32* (37), 6503. https://doi.org/10.1088/0305-4470/32/37/302.

(19) Meroz, Y.; Sokolov, I. M.; Klafter, J. Test for Determining a Subdiffusive Model in Ergodic Systems from Single Trajectories. *Phys Rev Lett* **2013**, *110* (9). https://doi.org/10.1103/PhysRevLett.110.090601.
